# Phenotypic plasticity of natural *Populus trichocarpa* populations in response to temporally environmental change in a common garden

**DOI:** 10.1186/s12862-019-1553-6

**Published:** 2019-12-26

**Authors:** Yang Liu, Yousry A. El-Kassaby

**Affiliations:** Department of Forest and Conservation Sciences, The University of British Columbia, 2424 Main Mall, Vancouver, British Columbia V6T 1Z4 Canada

**Keywords:** Phenotypic plasticity, Local adaptation, Natural selection, Common-garden approach, Climate change, Ordinary least-squares, *Populus trichocarpa*

## Abstract

**Background:**

Natural selection on fitness-related traits can be temporally heterogeneous among populations. As climate changes, understanding population-level responses is of scientific and practical importance. We examined 18 phenotypic traits associated with phenology, biomass, and ecophysiology in 403 individuals of natural *Populus trichocarpa* populations, growing in a common garden.

**Results:**

Compared with tree origin settings, propagules likely underwent drought exposures in the common garden due to significantly low rainfall during the years of measurement. All study traits showed population differentiation reflecting adaptive responses due to local genetic adaptation. Phenology and biomass traits were strongly under selection and showed plastic responses between years, co-varying with latitude. While phenological events (e.g., bud set and growth period) and biomass were under positive directional selection, post-bud set period, particularly from final bud set to the onset of leaf drop, was selected against. With one exception to water-use efficiency, ecophysiology traits were under negative directional selection. Moreover, extended phenological events jointly evolved with source niches under increased temperature and decreased rainfall exposures. High biomass coevolved with climatic niches of high temperature; low rainfall promoted high photosynthetic rates evolution.

**Conclusions:**

This work underpins that *P. trichocarpa* is likely to experience increased fitness (height gain) by evolving toward extended bud set and growth period, abbreviated post-bud set period, and increased drought resistance, potentially constituting a powerful mechanism for long-lived tree species in surviving unpredictably environmental extremes (e.g., drought).

## Background

Anthropogenic climate change is rapidly altering the environments experienced by flora and fauna [1, 2]. Widespread tree dieback events and other large-scale disturbances are already underway in many forests and woodlands [3–5]. These trends have been attributed to the direct and indirect impacts of drought stress and elevated temperatures and are expected to continue as a result of further global warming and drying [3, 5–7]. Under high environmental variability, natural selection is thought to favor flexibility in the form of phenotypic plasticity (i.e., the capacity of a genotype to render different phenotypes under variable environments; [8, 9]). Phenotypic plasticity can affect evolution by shifting phenotypes that are under natural selection [8]. Moderate levels of plasticity can promote evolution by, for example, hastening adaptation to an altered environment by increasing the frequency of beneficial alleles; on the other hand, strong plasticity could impede population responses to natural selection by removing selective forces that would otherwise drive adaptation through genetic change [10–12]. If patterns of plasticity are genetically variable, phenotypic plasticity can evolve in response to selection imposed by variable environments [13–15]. One of the essential goals in understanding the impact of climate change on plants is to determine the relative contribution to population persistence made by phenotypic plasticity, phenotypic selection, and the evolution of the plastic response in fitness-related traits.

Common-gardens are perhaps the most powerful approach to explore the goal of how climate change likely affects plasticity. In a common garden, propagules from natural populations are reared under the same condition; environmental factors associated with population differentiation can be evaluated based on the strength of genetic clines in phenotypes, assuming that average phenotypes vary due to natural selection and reflect local adaptation to environmental gradients [16]. Given a common environmental exposure, thus over time phenotypic differences can be ascribed to genetic differences rather than the effect of ontogeny or plastic developmental responses. In general, geographically separated populations have undergone bouts of selection imposed by their biotic and abiotic factors, leading to different genetic population structure. Hence, a common-garden experiment permits testing the predictions of adaptation theory (e.g., [17–19]) and revealing the genetic differentiation among populations as set by their past environments (e.g., a burgeoning field of genome-wide association studies).

We invoked common-garden experiments to assess adaptability under temporally environmental changes by using black cottonwood (*Populus trichocarpa*) natural populations as a study system. Rapid anthropogenic global change is jeopardizing the persistence of populations and species, particularly trees that define the ecosystem they occupy and shape local biodiversity [20, 21]. However, a paucity of studies has dealt with this question in forest trees (e.g., [22–24]). The genetic basis of phenology traits (e.g., bud-break [25]), biomass traits (e.g., wood characteristics [26]) and ecophysiology traits (e.g., stomata [27]) in *P. trichocarpa* has been detected and underpins population-wide geographical patterns. Genetically based clines for these traits can signify adaptation to continuous environmental variation along gradients, yet phenotypic divergence along gradients can also arise due to plasticity and/or neutral processes [28, 29].

To determine whether trees genetic clines are adaptive over time, 18 phenology, biomass, and ecophysiology traits of 403 *P. trichocarpa* genotypes from 29 natural populations were assessed in a common garden over multiple years. In this study, we first asked whether patterns of within-generation phenotypic plasticity of each trait differed between years and varied across populations as well as the relationship between plasticity and fitness. Second, we studied the extent of the temporal variation in the form, direction, and magnitude of natural selection imposed on phenotypic traits. Addressing these two questions informs us whether plasticity of the studied traits is adaptive or not. If plasticity can increase relative fitness across variable environments, then plasticity is adaptive; conversely, plasticity is nonadaptive if it moves phenotypes farther away from the optimum due to environmental changes [12, 30]. Moreover, non-adaptive plasticity can also be neutral due to differences in trait expression through development. Third, we investigated whether traits under strong selection exhibit more pronounced joint evolution with source niche climate. Linking responses of populations to environmental variation and the conditions to which they evolved in the past helps predict possible tendencies of trait evolution under climate change.

## Results

Main results would be reported based on the analysis of 18 traits related with phenology, biomass, and ecophysiology in 403 individuals from 29 natural populations of *Populus trichocarpa*, measured in a common garden over consecutive years, 2008–2010.

### Propagules in the common garden likely underwent drought exposures

**Fig. 1:**
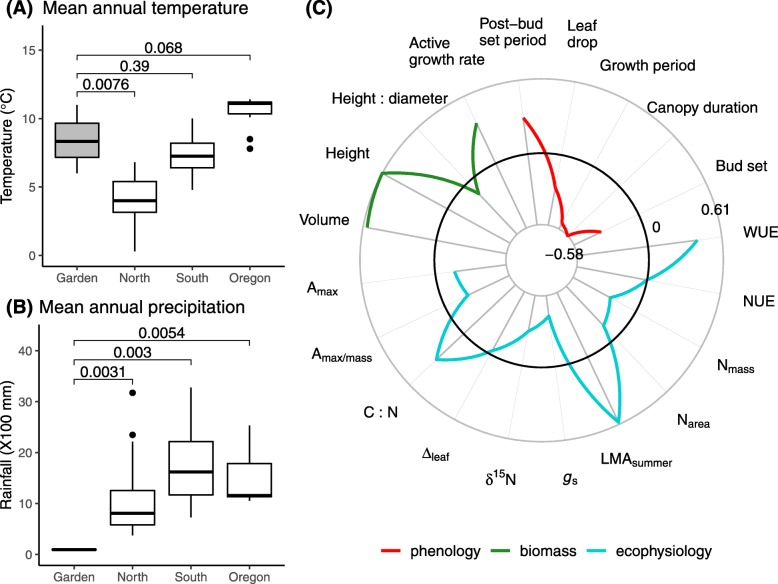
Common garden climates over data collection periods contrasting with historical climates of tree origins and their corrections with the traits. Comparisons of mean annual temperature (MAT; **A**) and mean annual precipitation (MAP; **B**) between a common garden and tree origin sites of three demes (North, South and Oregon) with *P*-values shown based on Wilcoxon test. We used MAT and MAP over years 2008–2010 for propagules growing in the common garden and 30 years (1961–1990 normals) for the original natural sites (data extraction detailed in Additional file 1: Note S2). (**C**) Radar plot showing all trait-climate correlations in the common garden settings. All traits are grouped into three categories (phenology, biomass, and ecophysiology). The position of traits along the radial axis indicates the strength of correlation. The solid black line represents zero correlation; the region inside (or outside) this line represents negative (or positive) correlations, respectively. The maximum negative and positive correlations are − 0.58 and 0.61, respectively, marked on the graph. See Table 1 for concise annotation for the traits

Given all trait measurements performed prior to August of each year (Additional file 1: Note S1), climate in 2009 (or 2010) denoted the period of September 2008 (or 2009) to August 2009 (or 2010), respectively (data source in Additional file 1: Note S2). While monthly average temperature between the two years fluctuated mainly in January through March with a mean difference of 2 °C (Additional file 1: Figure S2A), considerable monthly precipitation changes occurred in September through March with a mean difference of 41 mm (Additional file 1: Figure S2A). Year 2010 had both higher mean temperature and more rainfall than 2009, but the difference was not statistically significant (*P* = 0.81 and 0.32, respectively, by paired *t*-test; Additional file 1: Figure S2B). By comparing climates in the common garden with in tree origin sites, we found that mean annual temperature in the Garden was significantly higher than in the origin sites of North (*P* = 0.0076 by Wilcoxon test; Fig. 1a) but not significantly different to sites of South and Oregon (*P* = 0.39 and 0.07, respectively; Fig. 1a); by contrast, mean annual precipitation was significantly lower in the Garden than in all tree origin sites of the three demes (all *P* < 0.05; Fig. 1b). This indicates that propagules in the common garden were likely to experience environmental exposures to drought.

Correlations between garden climate and traits showed negative relationships in all phenology traits but post-bud set period, all ecophysiology traits but C: N, LMA_summer_ and WUE, and positive relationships in all biomass traits but height: volume (Fig. 1c). Climate had high correlations (|ρ| > 0.4 and *P* < 0.001) positively with height (0.61), volume (0.57), LMA_summer_ (0.59), and WUE (0.41) and negatively with canopy duration (− 0.58), growth period (− 0.53), and *g*_s_ (− 0.42) (Fig. 1c). This indicates that differences in the expression of traits across years are associated with the environment that propagules experienced in the common garden.

### Population-by-year interaction reveals strength and direction of plasticity

**Fig. 2:**
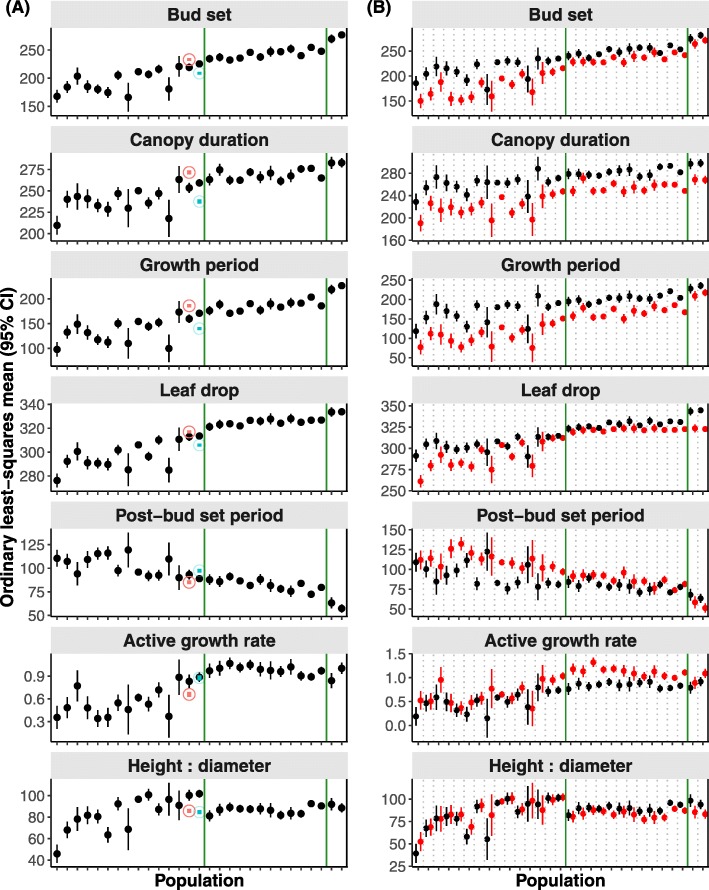
Population and temporal environment affect phenology and biomass traits independently and through their interactions. (**A)** Both population and temporal environment affect the traits. The graph shows ordinary least-squares (OLS) mean trait values for each population (black filled circle) and for two years representing two temporal environments (red and blue empty-circles for 2009 and 2010, respectively) from a REML-linear mixed model (Table 2). Hence, black circles show the mean trait values for each population (averaged over years); red and blue circles show the mean trait value (average across all populations) in 2009 and 2010, respectively. Note that the horizontal position of the red/ blue circles is uninformative and they are just placed in the center of each panel for visualization. Error bars represent 95% credible intervals and two vertical green lines delimit the populations into three demes, that is, northern, southern and Oregon (from left to right), where populations were aligned accordingly from 1 to 30 (details in Additional file 1: Figure S1 and Table S1). (**B**) Phenotypical plasticity between years is variable among populations. Populations are separated by vertical dashed lines. The filled-circles are OLS mean trait values for each population over two years (black and red dots for 2009 and 2010, respectively) with 95% credible intervals. See Table 1 for concise annotation for the traits

All five phenology and two biomass traits (Table 1) significantly varied among populations (all *P* < 0.001) and between years (all *P* < 0.001 except for height: diameter) (Fig. 2 and Table 2), reflecting population divergence likely due to local genetic adaptation and phenotypic plasticity. Our data revealed that genetic correlations between phenology traits were very strong (all |*r*| > 0.9, *P* < 0.001) in populations (Additional file 1: Table S2) and post-bud set period was negatively correlated with the other four phenology and two biomass traits (Additional file 1: Table S2). The average trait values clinally varied (Fig. 2a) in populations distributed in a latitudinal gradient (Additional file 1: Figure S1), indicative of genetically based clines. The populations of the northern deme had higher variability than those from the other two demes (Fig. 2a). Best linear unbiased predictions (BLUPs) can be used to compare which genotypes are more or less plastic [31] as shown in Additional file 1: Figure S3, and the plasticity differences were significant among genotypes based on tests for the random effect (i.e., ‘Genetics’) in all traits (all *P* ≤ 0.008) except for canopy duration (Table 2). The magnitude of the year effect was comparable to the variation ascribed to populations (i.e., value range of year-effect fell within population variation; Fig. 2a). For the phenology traits, only post-bud set period was higher in 2010 than in 2009, while the other traits had higher values in 2009 (Fig. 2A); for the biomass traits, active growth rate was higher in 2010 than in 2009 and height: diameter was comparable between the two years (Fig. 2a). This indicates that the magnitude of plasticity was considerably high for populations between years. Consistent with this observed pattern of plasticity between years, estimated trait values were higher in 2010 only for post-bud set period and active growth rate (Additional file 1: Figure S4). Furthermore, we found significant population-by-year interactions (all *P* ≤ 0.004; Table 2), attesting to among-population differences in the plastic responses to yearly varying environments. Compared with 2009, plasticity decreased in 2010 in all phenology traits except for post-bud set period (Fig. 2b). Populations of the northern deme were more sensitive to temporal environmental fluctuations than other populations in southern or Oregon demes (Fig. 2b).

**Table 1:** List of study traits measured in 403 *Populus trichocarpa* individuals from 139 provenances classified into 29 populations of three demes in a common environment over two years

Category/ Trait	Unit
Phenology trait	
Bud set	d (Julian date)
Canopy duration (bud break to 100% leaf drop)	d (Julian date)
Growth period (bud break to final bud set)	d (Julian date)
Leaf drop	d (Julian date)
Post-bud set period (bud set to 100% leaf drop)	d (Julian date)
Biomass trait	
Active growth rate	cm d^−1^
Height: diameter (ratio)	cm: cm
Height	cm
Volume	cm^3^
Ecophysiology trait	
A_max_ (maximum photosynthetic rate)	μmol CO_2_ m^−2^ s^−1^
A_max/mass_ (photosynthetic rate per unit dry mass)	μmol CO_2_ g^−1^ s^− 1^
C: N (carbon to nitrogen ratio)	mg mg^− 1^
∆_leaf_ (leaf carbon isotope discrimination)	‰
δ^15^N (stable nitrogen isotope ratio)	‰
*g*_s_ (stomatal conductance)	mol H_2_O m^−2^ s^− 1^
LMA_summer_ (leaf mass per unit area in summer)	mg cm^− 2^
N_area_ (leaf nitrogen content per unit area)	mg mm^− 2^
N_mass_ (leaf nitrogen content per unit dry mass)	mg mg^− 1^
NUE (photosynthetic nitrogen-use efficiency)	μmol CO_2_ g^−1^ N s^− 1^
WUE (instantaneous water-use efficiency)	μmol CO_2_ mmol^− 1^ H_2_O

**Table 2:** Variance partitioning of phenology and biomass traits using REML-linear mixed models for the comparison of trait responses across 29 Populations over two years and Year × Population interactions

		Bud set			Canopy duration			Growth period			Leaf drop			Post-bud set period			Active growth rate			Height: diameter		
	N DF	D DF	*F*/ χ^2^	*P*	D DF	*F*/ χ^2^	*P*	D DF	*F*/ χ^2^	*P*	D DF	*F*/ χ^2^	*P*	D DF	*F*/ χ^2^	*P*	D DF	*F*/ χ^2^	*P*	D DF	*F*/ χ^2^	*P*
Popltn	28	582	44.02	**< 10**^**−4**^	509	21.44	**< 10**^**−4**^	547	36.11	**< 10**^**− 4**^	619	42.20	**< 10**^**− 4**^	497	23.464	**< 10**^**− 4**^	573	17.53	**< 10**^**− 4**^	609	13.03	**< 10**^**−4**^
Year	1	424	297.48	**< 10**^**−4**^	458	441.68	**< 10**^**−4**^	436	556.53	**< 10**^**−4**^	439	263.22	**< 10**^**−4**^	410	93.455	**< 10**^**−4**^	444	123.08	**< 10**^**−4**^	456	1.40	1
Popltn × Year	28	424	6.08	**< 10**^**−4**^	459	2.55	**0.004**	437	5.48	**< 10**^**−4**^	439	7.26	**< 10**^**−4**^	411	6.913	**< 10**^**−4**^	445	3.18	**< 10**^**−4**^	456	2.44	**0.001**
Genetics	1	NA	54.79	**< 10**^**−4**^	NA	6.31	0.12	NA	27.22	**< 10**^**−4**^	NA	92.109	**< 10**^**−4**^	NA	11.424	**0.008**	NA	45.39	**< 10**^**−4**^	NA	80.167	**< 10**^**−4**^
Model fit statistics	*R*^2^	0.878			0.735			0.846			0.894			0.714			0.763			0.718		
	AIC	6393.95			6381.67			6751.05			5372.29			6050.42			−42.16			5953.03		
	REML	6273.95			6261.67			6631.05			5252.29			5930.42			− 162.16			5833.03		

We report on *F*-values for fixed effects and on χ^2^-values for random effects (i.e., ‘Genetics’). Significance of random effects was assessed using likelihood ratio tests. Significant *P*-values after the sequential Bonferroni correction (α = 0.05) are shown in bold. Denominator degrees of freedom (D DF) are calculated using the Satterthwaite’s approximation as implemented in the R package lmerTest. Popltn: Population. Variance-partitioning for ecophysiology traits reported in Additional file 1: Table S3

In contrast to phenology and biomass traits, all ecophysiology traits significantly varied among populations (all *P* < 0.01), reflecting population differentiation for all traits; but only five of the 11 ecophysiology traits (C: N, δ^15^N, *g*_s_, LMA_summer_, and WUE) significantly differed between years (all *P* ≤ 0.033) (Additional file 1: Table S3 and Figure S5), indicating plasticity of the five ecophysiology traits. These traits were significantly correlated with at least another ecophysiology trait (all *P* < 0.05, smallest |*r*| = 0.63) except for δ^15^N and LMA_summer_ (Additional file 1: Table S2). The average trait values varied less clearly along a latitudinal gradient (Additional file 1: Figure S5) compared with phenology and biomass traits (Fig. 2). The populations of the northern deme displayed higher variability than those from the other two demes (Additional file 1: Figure S5). BLUPs seemed homogenous across the populations (Additional file 1: Figure S3) and the random effect (‘Genetics’) was significant only in ∆_leaf_ and *g*_s_ (all *P* ≤ 0.0005; Additional file 1: Table S3). The magnitude of population-level plasticity between years was minor compared to the variation due to population (Additional file 1: Figure S5). Moreover, there was no significant population-by-year interaction for all these traits except for A_max_ (Additional file 1: Table S3), indicating minor among-population differences in the plasticity of ecophysiology traits. Similar to phenology and biomass, populations of the northern deme were more sensitive to different temporal environments in ecophysiology (Additional file 1: Figure S6).

### Population differentiation in traits is driven by selection

**Fig. 3:**
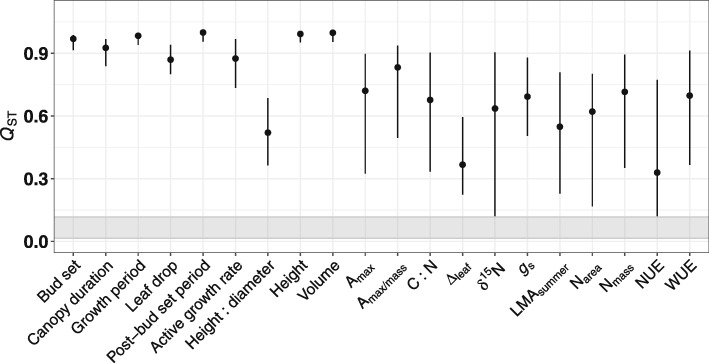
Estimate of quantitative trait differentiation (*Q*_ST_) among 29 populations. Circle marks point estimate and line indicates 95% Bayesian credible intervals. A grey band shows 95% credible intervals for global neutral *F*_ST_ among the populations. See Table 1 for concise annotation for the traits

The *Q*_ST_ analysis indicated high differentiation in the phenology, biomass, and most of ecophysiology traits (Table 1) among the 29 populations (Fig. 3). Both point estimates and 95% Bayesian CIs exceeded the *F*_ST_ 95% CI (0.015–0.177) for all traits but δ^15^N and NUE (Fig. 3). In particular, phenology and two biomass traits (height and volume) showed indications of considerably higher than neutral divergence (*Q*_ST_ > 0.8 > > *F*_ST_) (Fig. 3). This indicates that natural selection imposed on phenology and biomass prevailed over on ecophysiology. This additionally confirmed phenotypic divergence in the common garden as evidence of adaptation rather than drift.

### Strength and direction of selection on traits over time

In the phenotypic selection analysis, annual height gain was used as a proxy for fitness. Fitness had significantly high correlations with phenology traits (|ρ| ranging from 0.55 to 0.88; all *P* < 0.0001; Additional file 1: Figure S7). For biomass traits, fitness was highly correlated with active growth rate (ρ = 0.88, *P* < 0.0001) as expected (N.B. fitness estimated as plant size (height) gain here), but not with height: diameter (ρ = − 0.01, *P* = 0.1064) (Additional file 1: Figure S7). Nine of 11 ecophysiology traits had significant correlations (*P* < 0.05) with fitness, in which only C: N and WUE showed positive relationships (Additional file 1: Figure S7). Individuals with higher fitness also exhibited higher phenotypic plasticity for two phenology traits (bud set and growth period with ρ = 0.7 and 0.4, respectively; both *P* < 0.0001), active growth rate (ρ = 0.54; *P* < 0.0001), and three ecophysiology traits (C: N, NUE, and WUE with ρ = 0.18, 0.10, and 0.15, respectively; all *P* < 0.05) (Additional file 1: Figure S8), but there was a reverse fitness-plasticity relationship for post-bud set period (ρ = − 0.68; *P* < 0.0001), height: diameter (ρ = − 0.18; *P* < 0.0001), and two ecophysiology traits (δ^15^N and N_mass_ with ρ = − 0.16 and − 0.19, respectively; both *P* < 0.05) (Additional file 1: Figure S8).

Selection analysis revealed 36 cases of statistically significant selection: 24 cases of linear selection (β) on the 18 study traits, 11 cases of stabilizing (quadratic) selection (negative γ), and one case of disruptive selection (positive γ) (Table 3; statistic details in Additional file 1: Table S4 and S5). Similar results were obtained when relative fitness residuals were used as fitness (Additional file 1: Figures S9 and S10). With one exception to post-bud set period, high values of phenology and biomass traits (i.e., longer periods of phenological events and high biomass) were associated with increased fitness over two years (Table 3). On the premises of considering significant selection only, high ecophysiology trait values for WUE were associated with increased fitness consistently over the two years (Table 3); high ∆_leaf_ and *g*_s_ were consistently associated with lower fitness (Table 3). Based on the data pooled from both years, findings illustrated that linear selection on all 18 traits varied between years (Additional file 1: Table S6). With the exception of four ecophysiology traits (A_max/mass_, δ^15^N, *g*_s_ and N_area_), all tests for Year × β interactions for each trait rejected the null hypothesis that these selection differentials were equivalent (Additional file 1: Table S6), indicating that most of the traits had heterogeneous selection differentials between years. Specifically, selection direction was concordant over time for each of phenology and biomass traits (Table 3), but the magnitude of selection significantly differed over time. WUE and ∆_leaf_, though consistent selection directions, underwent different strength of selection over time (Table 3 and S6). In addition, high *g*_s_ was equally selected against over time (Table 3 and S6).

**Table 3:** Linear (β) and quadratic (γ) selection differentials for phenology, biomass, and ecophysiology traits

Trait	Year	β	γ
Bud set	2009	0.255***	− 0.043***
	2010	0.386***	−0.027
Canopy duration	2009	0.121***	−0.032
	2010	0.240***	−0.042
Growth period	2009	0.187***	−0.036*
	2010	0.347***	−0.102***
Leaf drop	2009	0.232***	−0.023
	2010	0.342***	0.201***
Post-bud set period	2009	−0.162***	−0.047**
	2010	−0.316***	−0.108***
Height: diameter	2009	0.082***	−0.060**
	2010	0.008***	−0.073*
Active growth rate	2009	0.293***	−0.032**
	2010	0.399***	−0.023
A_max_	2009	−0.077***	−0.024
	2010	0.002	−0.092
A_max/mass_	2009	−0.053**	−0.042*
	2010	−0.054	−0.038
C: N	2009	− 0.025	0.027
	2010	0.045	−0.065
∆_leaf_	2009	−0.054**	−0.050
	2010	−0.110***	−0.037
δ^15^N	2009	−0.002	−0.019
	2010	−0.028	−0.038
*g*_s_	2009	−0.093***	−0.035
	2010	−0.089**	−0.090*
LMA_summer_	2009	−0.037*	−0.018
	2010	0.027	−0.051
N_area_	2009	0.006	0.010
	2010	0.007	−0.046
N_mass_	2009	0.031	0.010
	2010	−0.059	−0.062
NUE	2009	−0.082***	−0.006
	2010	−0.011	−0.047
WUE	2009	0.029*	−0.007
	2010	0.097***	−0.093**

Significance: * *P* < 0.05, ** *P* < 0.01, *** *P* < 0.001

The signs and magnitudes indicate the direction and strength of the linear (β) or non-linear (γ) selection on each trait in either year. Significant *F*-tests indicates nonzero selection differentials (Additional file 1: Table S4) on a trait in a given year. The selection differential describes both direct and indirect selection on each trait. It equals to the regression coefficient of relative fitness onto standardized trait values after controlling the effect of any unmeasured traits on fitness by including a random intercept term, ‘Genetics’ (i.e., average Euclidean genetic distance using genetic marker data). For quadratic selection, a negative, significant value of γ indicates stabilizing selection, while a positive value is evidence for disruptive selection (tests detailed in Additional file 1: Table S5). Both selection analyses were visualized in Additional file 1: Figures S9 and S10

For thoroughness, we also calculated linear selection gradients to evaluate direct selection on each trait while controlling the effect of possible unmeasured traits on fitness (Additional file 1: Note S4). The selection gradients partly agreed with the selection differentials, and consistently showed that active growth rate was under positive selection over time (Additional file 1: Table S7). As the selection gradient determines statistically how much selection acts directly on each trait versus indirectly through correlations with other traits, missing data of ecophysiology traits may impede the reliability of such multivariate analysis applicable to our case. We therefore focused on analysis of selection differentials for results interpretation.

Finally, we presented evidence that stabilizing selection acted on height: diameter over the two years (Table 3 and Additional file 1: Figure S10; based on test for significance and peak falling within the values of a given trait across populations); also on traits in a single year, which included growth period, post-bud set period, A_max/mass_, *g*_s_ and WUE (Table 3 and Additional file 1: Figure S10). In addition, we found evidence of disruptive selection acted on leaf drop in one year (Table 3 and Additional file 1: Figure S10).

### Joint evolution of traits and source niche climate

**Fig. 4:**
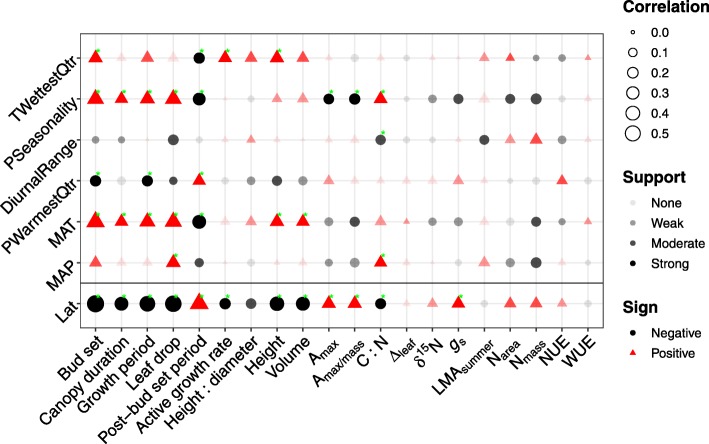
Correlated evolution analysis between trait and niche climate. Correlations are either positive (red triangle) or negative (black circle), vary in strength (size of shapes), and have different levels of support indicated by transparency of circle colors (none of support: log*BF* < 2, complete transparency; weak support: log*BF* > 2, high transparency; moderate support: log*BF* > 5, low transparency; strong support: log*BF* > 10, no transparency). Green asterisks indicate correlations that meet the criterion threshold adjusted for multiple comparisons. After a reduction of collinearity among climatic variables, six of 19 bioclimatic variables were selected and visualized in an ascending order (top-down) based on relative contribution to niche suitability (details in Additional file 1: Note S8). Climate variables and their abbreviations: Mean Annual Temperature (MAT), Mean Diurnal Range (DiurnalRange), Mean Temperature of Wettest Quarter (TWettestQtr), Mean Annual Precipitation (MAP), Precipitation Seasonality (PSeasonality) and Precipitation of Warmest Quarter (PWarmestQtr). See Table 1 for concise annotation for the traits

We investigated evolutionary associations between traits and the environment that is tied to source niche climate conditions populations were exposed to in the past. To begin with, phenology and biomass traits coevolved with latitude (Fig. 4), which supported these traits variation in a latitudinal gradient (Fig. 2a). Phenology traits had most strongly supported evolutionary associations with niche climates. Specifically, four of five phenology traits (bud set, canopy duration, growth period, and leaf drop) had positive associations with both temperature (MAT) and rainfall (PSeasonality); post-bud set period was also highly associated with these two climate variables but in a negative manner (Fig. 4). Moreover, increased temperature in wettest quarter (TWettestQtr) and decreased precipitation in warmest quarter (PWarmestQtr) promoted phenology evolution towards extended bud set and abbreviated post-bud set period (Fig. 4). High investment in biomass (height and volume) was found in areas with high temperature (Fig. 4). In addition, high photosynthetic rate (A_max_ and A_max/mass_) had strongly supported negative associations with precipitation (PSeasonality); by contrast, high rainfall (MAT and PSeasonality) was strongly associated with C: N in a positive manner (Fig. 4). However, WUE was not significantly driven by any climate variable of source niches (Fig. 4). Estimated correlation coefficients in all the BayesTraits analyses ranged from − 0.33 to 0.41, and 24 of 120 were individually strongly supported (log*BF* > 10). The 19 additional correlations were moderately supported (10 ≥ log*BF* > 5); 28 had weak support (5 ≥ log*BF* > 2), and the remaining 49 lacked substantial support (log*BF* ≤ 2). Around one-fifth (24 out of 120) of the comparisons had a Bayes factor that exceeded the threshold of 9.8 to hold across all comparisons (Fig. 4). Finally, we note that a species distribution model (unpubl. Work; Additional file 1: Figure S11) showed that Mean Annual Precipitation and Mean Annual Temperature contributed 40 and 39% to the model prediction, respectively; PSeasonality only accounted for 5.5% (more detail in Additional file 1: Note S8). This indicates that climate variables key to population persistence may not directly drive trait evolution.

## Discussion

This study used common-gardens to study the contemporary evolution of a species to climate change and linked trait evolution and source niche climate to discern the joint evolution of traits with climatic niches in a macroevolutionary context. This study revealed that *P. trichocarpa* is likely to cope with environmental changes (e.g., extreme climates of drought) through the evolution of extended bud set and growth period, abbreviated post-bud set period, and increased drought resistance in ecophysiology.

### Patterns of phenotypic plasticity and its adaptability

In *P. trichocarpa*, population and temporal environment interacted to affect the strength and direction of plasticity in phenology and biomass traits. All five phenology and two biomass traits had significant differences among populations and between years with substantial population-by-year interactions (Table 2 and Fig. 2a), highlighting the consequences of phenology and biomass changes in response to environmental differences. This corroborates relatively high plasticity in phenological events [32–34] and biomass allocation traits [35] in other woody species. By contrast, ecophysiology traits exhibited significant disparities between populations (Additional file 1: Table S3 and Figure S5), in support of genetic differentiation among populations. This was in agreement with previous findings in, for instance, A_max_, ∆_leaf_, *g*_s_, N_mass_, and WUE [36–38]. However, not all ecophysiology traits showed significant plasticity and differed between years among populations (Additional file 1: Table S3). This may be caused by biochemical and biophysical processes as responses to environmental conditions, largely influencing some ecophysiology traits, for instance, A_max_ [39] and ∆_leaf_ [40, 41]. Moreover, altering short-term ecophysiological processes may present an acclimation to environmental variation – a facultative and reversible form of plasticity [42, 43].

Natural selection drives the evolution of phenotypic plasticity such that there must be genetic variation in plasticity on which selection can act [44], leading to plasticity clines akin to genetically based counterparts. We found that populations in the northern deme were more plastic than those from the other two demes, southern or Oregon (Fig. 1B and Additional file 1: Figure S5). Possibly because the northern populations are more plastic in response to forcing (warm spring temperatures) or have larger geographic differences in the photoperiod and forcing interactions or the chilling (intensity of winter temperature) and forcing requirements in the common garden than southern or Oregon populations. Based on phenology and biomass traits, populations had elevated or dampened mean trait values roughly in a latitude gradient (Fig. 2a). This is congruent with the evidence that more variable environments (e.g., shorter photosynthetic periods and harsh winters for the northern deme) facilitate the evolution of greater plasticity when environmental cues are predictable [45–47]. Furthermore, this divergence between northern and the other demes may be genetically arisen by reproductive isolation due to a natural barrier – no cottonwood belt [48], limiting gene flow between the two deme groups. Additionally, the studied populations were separated by topographic barriers and habitat for each population may be heterogeneous, possibly leading to the exposure to different selective pressures.

In addition to the observed genetic variation in plasticity, significant correlations between plasticity and fitness constitute another important component for the evolvability of plasticity [49]. If plasticity can increase fitness in a new environment, then increased plasticity is expected to evolve; decreased plasticity might also evolve, if nonadaptive plasticity results in fitness decline [49]. Significant plasticity (i.e., G × E interactions in Table 2 and Additional file 1: Table S3) and fitness-plasticity relationships (Additional file 1: Figure S8) indicate that increased plasticity in bud set, growth period, active growth rate, and WUE was likely to evolve; decreased plasticity in post-bud set period might coevolve as well due to its strong negative correlation with bud set (ρ = − 0.99, *P* < 0.0001; Additional file 1: Table S2).

### Evolutionary potential for plasticity driven by natural selection

Individuals that can modify their development in different environments must be endowed with the ability to obtain high fitness. Temporal variation in fitness response to environmental changes quantifies the extent of temporal heterogeneity of selection, providing clues to better assess the cumulative patterns of adaptive variation over time [50–52]. With one exception to post-bud set period, phenology traits were under significant positive selection (Table 3). This indicates that selection consistently favored extended durations of bud set, canopy (bud break to leaf drop), growth (bud break to final bud set) and leaf drop while disfavoring long post-bud set period (bud set to leaf drop), or rather, selecting for a shorter period between final bud set and timing of leaf drop. Contradictory to the selection pattern of WUE, most ecophysiology traits (e.g., ∆_leaf_ and *g*_s_) were under significant negative selection (Table 3). Moreover, active growth rate and height: diameter were selected for over the two years (Table 3). It suggests that *P. trichocarpa* evolved toward increasing biomass accumulation (fitness gain) through extended canopy duration while abbreviated duration from final bud set to timing of leaf drop, which would occur through increased WUE to escape drought or improve drought tolerance [53, 54]. This outcome supported that drought pressures were likely imposed on the propagules in the common garden (Fig. 1). Similar to the common-garden experiment, a resurrection study in an annual plant also documented rapid evolutionary changes in traits related to flowering phenology, drought tolerance (WUE), and reproductive fitness [55].

Furthermore, the selection analysis showed that given significant selections, all selection directions were consistent over time for single traits (Table 3). Without considering tests of significance, there were eight of 36 cases of selection whose direction reversed sign between years (Table 3). This supports temporal fluctuations were primarily found in traits that underwent relatively weak selection [51], with sampling errors inflating heterogeneity in most estimates of selection [56]. Fluctuating selection can lead to evolutionary stasis if the direction of selection reverses sign frequently [51, 57]. These temporal changes in the sign of directional selection could manifest in stabilizing selection across multiple episodes of selection as populations repeatedly traverse the summit of a fitness peak [58, 59]. Evolutionary stasis arises if the position of the peak within phenotypic space is stable [60]. Nonetheless, long-term studies are needed to test whether temporal variation in selection could account for the stability of genetic clines, although short-term directional selection is very frequent. In addition, we detected evidence for stabilizing selection in seven of the eight significant quadratic selection on traits, that is, selection in favor of intermediate trait values (Table 3 and Additional file 1: Figure S10; based on significance test and peak falling within data range).

### Joint evolution of phenotypic traits with climatic niches

Studying the joint evolution of traits with climate in a macroevolutionary context allows us to know historically evolutionary drivers and directions of traits and thus to better predict the impact of environmental changes on trait evolution. The tendency for post-bud set period to be longer for plants from warmer climates (Fig. 4) is consistent with countergradient variation [61], while the other phenology traits showed cogradient variation (Fig. 4). Comparative analyses of niche macroevolution in plants have revealed that the evolution of some key traits (e.g., leaf forms and phenology) can facilitate the colonization of stressful environments within arid or cold climates [62, 63]. In this study, high genetic correlations among phenology and biomass traits prompt joint evolution of these traits driven by similar evolutionary forces (Additional file 1: Table S2). We confirmed that both phenology and biomass traits were driven by common climate variables (e.g., MAT; Fig. 4) and respectively distinct climates (e.g., PSeasonality for phenology and TWettestQtr for biomass; Fig. 4). Of these, Mean Annual Temperature and Precipitation Seasonality were most important in influencing traits (Fig. 4); the former was found to considerably contribute to species distribution (39%) based on a species distribution modeling (Additional file 1: Figure S11 and Note S7; unpubl. work). However, Mean Annual Precipitation had a substantial contribution to species distribution (40%) but played a less important role in the evolution of studied traits, except for leaf drop and C: N (Fig. 4).

### Caveats and limitations

To examine phenotypic plasticity in plants, multiple common gardens with different environmental conditions are usually established, where clones are planted. Such an experimental design permits assessing plastic responses of individuals with the same genetic makeup but exposed to differential environments at the same time [16]. For long-lived trees, a workable approach is to cultivate clonal ramets in greenhouse and then outplant propagules in common gardens; before propagules are well acclimated to field planting sites for trait measurements, additional 5–6 years are needed, in addition to propagules’ production (e.g., [64–66] for common garden approaches used in forest trees). However, there remain potential issues in testing plasticity for those propagules: ontogeny confounded with plasticity; microbiome interacting with developing roots; moreover, as plant ages, decreased growth rate and increased leaf mass per unit area (LMA) due to decreased foliar biomass can be expected. All these factors should be considered and teased apart from plastic responses as best one can, for example, modeling with plant development terms included.

This large-scale study reasonably allows plasticity testing based on temporally environmental changes in a common garden, on the grounds that the study spanned two consecutive years using branch cuttings rooted for 9–10 years and propagules were exposed to stronger environmental pressures over the two years compared with the settings of tree origins (Fig. 1b); moreover, there were obviously environmental fluctuations between both years (Additional file 1: Figure S2). We additionally found that the expression of traits over the two years were highly correlated with the environmental conditions of the common garden (Fig. 1c). Altogether, these results point to plastic responses to environmental conditions instead of ontogeny effects playing a main role in changes in expressed traits over time.

## Conclusions

While genetic clines in traits of ecological relevance are relatively fixed, changing environmental conditions may alter the shape of these clines by imposing novel selection. Understanding the magnitude and direction of phenotypic plasticity and selection permits predicting the evolutionary potential for the adaptability of plasticity to environmental changes; further combining with trait evolution jointly with climatic niches ascertains possible long-term tendencies of trait evolution under climate change. In general, natural selection imposed on phenology and biomass traits prevailed over on ecophysiology counterparts (Fig. 3). Trait- or plasticity-fitness relationships (Additional file 1: Figures S7 and S8) and selection analysis (Table 3) consistently showed that plasticity of bud set, growth period, active growth rate, and WUE is adaptive (significant positive selection and strong positive correlation between plasticity and relative fitness) over time, in contrast to that of post-bud set period in a nonadaptive direction (negative selection and plasticity-fitness correlation). Analysis for the joint evolution of traits with source niche climate illustrated that bud set and growth period were strongly driven by MAT and PSeasonality in a positive direction, contrasting to strong negative correlations with post-bud set period (Fig. 4). Overall, we presented evidence that *P. trichocarpa* is able to increase fitness via increasing active growth rate (biomass) and likely to extend bud set and entire growth period as responses to less limiting temperature (including less frequent frost events) due to climate change, reconciled by abbreviating the duration from final bud set to the onset of leaf drop, and increase drought resistance via increasing WUE. Therefore, this study shows evidence that phenotypic plasticity likely represents a vital flexibility that long-lived trees could use to adapt to rapidly changing environments.

## Methods

### Study system and common garden

Branch cuttings were collected from naturally growing trees of *Populus trichocarpa* Torr. & A. Gray by British Columbia Ministry of Forests, Lands and Natural Resource Operations [48]. These trees were located in 29 drainages (topographic units separated by watershed barriers) extending 14° in latitude (45.6–59.6°N) spanning the species’ geographic distribution range (44 to 60°N, − 121 to − 138°W) in the Pacific Northwest (Additional file 1: Figure S1). Cuttings were rooted and outplanted at Surrey, British Columbia, Canada (49.19°N, − 122.85°W, 134 masl) in 2000. In spring 2008, propagules were collected from the Surrey site and used to establish a randomized, replicated common garden at the University of British Columbia Research Totem Field (49.26°N, − 123.25°W, 82 masl) [67]. Field assessments of a suite of traits in the common garden were conducted in three consecutive years (N.B. 2008 only for height) for 403 *P. trichocarpa* individuals, and each individual was replicated by 4–20 clonal ramets. These individuals were grouped into 139 provenances within the 29 drainages (populations, hereafter) and based on genetic population structure deciphered by genetic markers [68], they belong to three demes (i.e., northern, southern, and Oregon (southernmost); Additional file 1: Table S1 and Figure S1).

### Trait measurements

The measurements of phenology, biomass, and ecophysiology traits have previously been described ([69]; also see Additional file 1: Note S1 and Table 1 for concise annotation). Briefly, seasonal canopy events were recorded directly from observations of trees and calculated additional traits based on phenological date information. The Julian dates of phenological events were recorded for each tree, including bud break, final bud set and leaf drop. Phenology events were marked using visual observations of the terminal bud on the main bole or canopy as a whole. General growth was estimated at the end of each season by measuring tree height and basal diameter at 10 cm from the ground. Active growth rate was determined from yearly height gain divided by the growth period. All ecophysiology traits measurements assessed by gas exchange using either a LI-COR 6400 or LI-COR 6400 XT portable infrared gas exchange system (LI-COR Biosciences). Three gas exchange traits were directly measured including maximum photosynthetic rate (A_max_), stomatal conductance (*g*_s_), and instantaneous water-use efficiency (WUE) as determined by photosynthetic rate over transpiration under constant vapor pressure deficit. After gas exchange sampling, two leaf tissue discs were taken using a standard, hand-held punch from an upper canopy leaf on each tree. Samples were oven dried at 50 °C for 48 h and weighed to determine leaf mass per unit area (LMA) and to calculate photosynthetic rate per unit dry mass (A_max/mass_). Between 2 and 2.5 mg of dried tissue was analyzed for carbon (C) and nitrogen (N) content and stable isotope ratios (δ^13^C and δ^15^N, respectively). Based on these data, C to N ratio (C: N), leaf N content per unit area (N_area_) and per unit dry mass (N_mass_) and photosynthetic N-use efficiency (NUE). δ^13^C values were used with correction for sampling date to obtain net discrimination (∆_leaf_) as a proxy measurement for time-integrated WUE.

### Statistical analysis

All statistical analyses were carried out in R v.3.5.1 [70]. All *P*-values throughout this study were adjusted using the sequential Bonferroni correction [71]. Additional details of this section are available in the Supplemental Methods (Additional file 1: Notes S3-S8).

#### Partitioning variance at population and temporal levels

We utilized univariate REML-linear mixed models (LMMs) to partition population and environment contributions to the variation in each of the 18 study traits (type III ANOVA using the R packages lme4 and lmerTest [72, 73];). We specified separate models for each trait with fixed effects including Year of measurement in a common garden (Year), Population (nested in Year), and their interactions (more details in Additional file 1: Note S3). A significant Year effect indicates plastic responses to environmental conditions (i.e., trait plasticity) and a significant Population effect indicates population-level differences in traits (i.e., genetic differentiation). Year × Population interaction implies plastic responses differ among populations (i.e., different plasticity among populations reflected by G × E interactions [74];). Moreover, we included a random factor, ‘Genetics’, estimated by the average Euclidean genetic distance between genotypes using genetic marker (single nucleotide polymorphism) data. To quantify plasticity at the population level and between years, we estimated ordinary least-squares (OLS) mean trait values for the fixed effects. We extracted OLS values for a specific fixed effect from the LMM by using the ‘lsmean’ function of the R package lsmeans [75]. OLS mean trait values for Year, Population, and Year × Population effects were used to quantify plasticity. In addition, we performed Spearman’s rank correlation tests of the OLS means for the Population fixed effect to verify the correlations of the traits.

#### Q_ST_-F_ST_ comparisons

To test the hypothesis that the study traits differentiation among populations is driven by natural selection, we estimated levels of trait divergence based on *Q*_ST_ – the quantitative genetic equivalent of *F*_ST_ [76, 77]. *Q*_ST_ was estimated following the method of [78]. The additive variance components between (${\sigma}_{between}^2$) and within (${\sigma}_{within}^2$) populations for each trait were obtained through nested ANOVA (i.e., provenances nested within populations) using the Markov chain Monte Carlo (MCMC) approach in the R package MCMCglmm [79]. In a Bayesian framework, each model employed resampling strategies across individuals within population and specifically, we used inverse Wishart priors and an MCMC of 50,000 iterations with a burn-in of 10%. Some traits were log or square root transformed to improve homoscedasticity. *Q*_ST_ was calculated as ${\sigma}_{between}^2$ / (${\sigma}_{between}^2$ + $2{\sigma}_{within}^2$) and pairwise neutral *F*_ST_ estimates for the 29 populations had been calculated [68]. If *Q*_ST_ value significantly exceeds *F*_ST_, divergent selection can be inferred, whereas the null hypothesis that phenotypic differences are consistent with drift alone cannot be rejected if *Q*_ST_ does not differ significantly from *F*_ST_ [76, 77, 80].

#### Phenotypic selection analysis

Prior to selection analysis, fitness was graphed against plasticity of each trait across populations. The fitness-plasticity relationship simply showcased whether plasticity of a given trait was in an adaptive direction. Here, the plasticity of a genotype was calculated as the mean difference in trait values between the two years [81] and annual height gain was used as a proxy for fitness. Selection analyses [82] were then performed in each year using linear mixed models to assess whether changes in phenology, biomass, and ecophysiology traits were adaptive and followed directional selection. Standardized linear (β) and quadratic (γ) selection differentials were estimated as the regression coefficients of relative fitness on the standardized mean trait values of genotypes in each year [83]. Our objective was to estimate the influence of a fitness function likely to vary between populations, and shifting over time, and thus we relativized fitness and standardized trait values (Z-scores) [84]. Moreover, these models included a random intercept ‘Genetics’ term to control possible effects of unmeasured traits on fitness. Additionally, we tested whether directional selection on each trait was temporally heterogeneous by pooling data from both years and then fitting a mixed-effects ANCOVA model with an additional Year × Trait (β) interaction term (details in Additional file 1: Note S4). Separate linear and quadratic models were conducted for each year to retrieve selection differentials (β and γ) and *P*-values. The parameter estimate from the quadratic regressions were doubled to obtain the quadratic selection differentials (details in Additional file 1: Note S5) [85]. In all cases, significance was assessed by performing permutation tests with 5000 bootstrap samples. Finally, relative fitness residuals of all individuals in each year (Additional file 1: Note S6) were regressed onto standardized trait values in a linear or quadratic way such that the comparisons of trait selection analysis can be directly visualized between years.

#### Coevolution of traits with source niche climate

We tested the hypothesis that phenotypic trait evolution is driven by source niche climate using the program BayesTraits [86] implemented in the R wrapper package ‘btw’. This program analyzes continuous phenotypes using a phylogenetically generalized least-squares approach under the assumption of Brownian motion, estimating correlation coefficients and measures of support for correlated evolution between variables. A phylogenetic tree for the individuals was constructed using individual Euclidean genetic distance (Additional file 1: Figure S12; unpubl. work). For each set of phylogenetic tree and phenotypes or climate variables, we assessed a model using the continuous function under an MCMC setting, estimating the log marginal likelihood using the stepping stone method with 100 stones and 1000 iterations per stone. We estimated the log Bayes factor (log*BF*) for the dependent model (correlation between variables allowed) against the independent model (all correlations fixed to zero) as twice the difference between the estimated log marginal likelihoods. We interpreted comparisons where log*BF* > 2, 5 or 10 as having weak, moderate, or strong support, respectively (see Additional file 1: Note S7 and S8 for more details).

## Supplementary information

**Additional file 1.** **Table S1.** Information about the study populations. **Table S2.** Pairwise Spearman’s rank correlations for the trait genetic relationship among the 29 populations calculated using population-level ordinary least-squares mean trait values. **Table S3.** Statistics from REML-linear mixed models of ecophysiology traits for the comparison of trait responses across 29 Populations in two years (i.e. temporal Environments) and Year × Population interactions. **Table S4.** Linear phenotypic selection analysis (selection differentials) of the 18 traits over two years. **Table S5.** Non-linear (quadratic) selection differentials of the 18 traits over two years. **Table S6.** Test for heterogeneity of directional selection on the 18 traits between years. **Table S7.** Linear phenotypic selection gradients of the 18 traits over two years. **Figure S1.** 29 natural *Populus trichocarpa* populations marked on the map with this species’ geographic distribution range shaded in grey. **Figure S2.** Climate of the common garden over the trait measurement periods 2008-2010. **Figure S3.** Best linear unbiased predictions (BLUPs) for each genotype by trait. **Figure S4.** Estimated phenology and biomass trait values for each genotype compared by year. **Figure S5.** Population and temporal environment affects ecophysiology traits independently. **Figure S6.** Population and temporal environment affects ecophysiology traits through their interactions. **Figure S7.** Correlation between standardized traits (Z-scores) and relative fitness (scaled height gain over single years). **Figure S8.** Fitness (height gain) and plasticity (reaction norm) relationship for the study traits. **Figure S9.** Visualization of linear selection differentials for each of the 18 traits. **Figure S10.** Visualization of quadratic selection differentials for each of the 18 traits. **Figure S11.** Present-day *P. trichocarpa* suitability scores across its distribution range. **Figure S12.** Individual phylogeny based on individual Euclidean genetic distance. **Note S1.** Trait measurements. **Note S2.** Climate data over the period of trait measurements (2008-2010). **Note S3.** REML-linear mixed model to partition variance in the 18 traits. **Note S4.** Estimating directional selection in each year. **Note S5.** Estimating quadratic selection in each year. **Note S6.** Estimating relative fitness residuals in each year.** Note S7.** Analysis of the joint evolution of traits and source niche climate. **Note S8.** Niche suitability identifying key climate variables for species persistence.

## Data Availability

All original data supporting the findings of this study have been made available at Dryad Digital Repository [87]. The original genotyping data of the POPCAN project have been deposited on SRA under the accession PRJNA276056.

## References

[1] Walther G-R, Post E, Convey P, Menzel A, Parmesan C, Beebee TJC, Fromentin J-M, Hoegh-Guldberg O, Bairlein F (2002). Ecological responses to recent climate change. Nature.

[2] Root TL, Price JT, Hall KR, Schneider SH, Rosenzweig C, Pounds JA (2003). Fingerprints of global warming on wild animals and plants. Nature.

[3] Allen Craig D., Breshears David D., McDowell Nate G. (2015). On underestimation of global vulnerability to tree mortality and forest die-off from hotter drought in the Anthropocene. Ecosphere.

[4] Midgley GF, Bond WJ (2015). Future of African terrestrial biodiversity and ecosystems under anthropogenic climate change. Nat Clim Chang.

[5] Anderegg WRL, Kane JM, Anderegg LDL (2013). Consequences of widespread tree mortality triggered by drought and temperature stress. Nat Clim Chang.

[6] Asner GP, Brodrick PG, Anderson CB, Vaughn N, Knapp DE, Martin RE (2016). Progressive forest canopy water loss during the 2012–2015 California drought. Proc Natl Acad Sci U S A.

[7] Cook BI, Smerdon JE, Seager R, Coats S (2014). Global warming and 21st century drying. Clim Dyn.

[8] Bradshaw AD. Evolutionary significance of phenotypic plasticity in plants. Adv Genet. 1965;13:115–55.

[9] Bradshaw AD (2006). Unravelling phenotypic plasticity -- why should we bother?. New Phytol.

[10] Baldwin JM (1896). A new factor in evolution. Am Nat.

[11] Price TD, Qvarnström A, Irwin DE (2003). The role of phenotypic plasticity in driving genetic evolution. P Roy Soc B Biol Sci.

[12] Ghalambor CK, McKay JK, Carroll SP, Reznick DN (2007). Adaptive versus non-adaptive phenotypic plasticity and the potential for contemporary adaptation in new environments. Funct Ecol.

[13] Sultan SE (2000). Phenotypic plasticity for plant development, function and life history. Trends Plant Sci.

[14] Hairston NG, Holtmeier CL, Lampert W, Weider LJ, Post DM, Fischer JM, Cáceres CE, Fox JA, Gaedke U (2001). Natural selection for grazer resistance to toxic cyanobacteria: evolution of phenotypic plasticity?. Evolution.

[15] Gomulkiewicz R, Kirkpatrick M (1992). Quantitative genetics and the evolution of reaction norms. Evolution.

[16] Aitken SN, Bemmels JB (2016). Time to get moving: assisted gene flow of forest trees. Evol Appl.

[17] Joshi J, Schmid B, Caldeira MC, Dimitrakopoulos PG, Good J, Harris R, Hector A, Huss-Danell K, Jumpponen A, Minns A (2001). Local adaptation enhances performance of common plant species. Ecol Lett.

[18] Angert AL, Schemske DW (2005). The evolution of species’ distributions: reciprocal transplants across the elevation ranges of *Mimulus cardinalis* and *M*. Evolution.

[19] Hereford J (2009). A quantitative survey of local adaptation and fitness trade-offs. Am Nat.

[20] Bellard C, Bertelsmeier C, Leadley P, Thuiller W, Courchamp F (2012). Impacts of climate change on the future of biodiversity. Ecol Lett.

[21] Garcia RA, Cabeza M, Rahbek C, Araújo MB (2014). Multiple dimensions of climate change and their implications for biodiversity. Science.

[22] Alberto FJ, Aitken SN, Alía R, González-Martínez SC, Hänninen H, Kremer A, Lefèvre F, Lenormand T, Yeaman S, Whetten R (2013). Potential for evolutionary responses to climate change - evidence from tree populations. Glob Change Biol.

[23] Wang T, Hamann A, Yanchuk A, O'Neill GA, Aitken SN (2006). Use of response functions in selecting lodgepole pine populations for future climates. Glob Change Biol..

[24] Matyas C (1994). Modeling climate change effects with provenance test data. Tree Physiol.

[25] McKown AD, Klápště J, Guy RD, El-Kassaby YA, Mansfield SD. Ecological genomics of variation in bud-break phenology and mechanisms of response to climate warming in *Populus trichocarpa*. New Phytol. 2018. 10.1111/nph.15273 29963703

[26] Porth I, Klápště J, Skyba O, Hannemann J, McKown AD, Guy RD, DiFazio SP, Muchero W, Ranjan P, Tuskan GA (2013). Genome-wide association mapping for wood characteristics in *Populus* identifies an array of candidate single nucleotide polymorphisms. New Phytol.

[27] McKown AD, Guy RD, Quamme L, Klápště J, La Mantia J, Constabel CP, El-Kassaby YA, Hamelin RC, Zifkin M, Azam MS (2014). Association genetics, geography and ecophysiology link stomatal patterning in *Populus trichocarpa* with carbon gain and disease resistance trade-offs. Mol Ecol.

[28] Montesinos-Navarro A, Wig J, Xavier Pico F, Tonsor SJ (2011). *Arabidopsis thaliana* populations show clinal variation in a climatic gradient associated with altitude. New Phytol.

[29] Kooyers NJ, Gage LR, Al-Lozi A, Olsen KM (2014). Aridity shapes cyanogenesis cline evolution in white clover (*Trifolium repens* L.). Mol Ecol.

[30] Ghalambor CK, Hoke KL, Ruell EW, Fischer EK, Reznick DN, Hughes KA (2015). Non-adaptive plasticity potentiates rapid adaptive evolution of gene expression in nature. Nature.

[31] Arnold PA, Kruuk LEB, Nicotra AB. How to analyse plant phenotypic plasticity in response to a changing climate. New Phytol. 2019;0(0). 10.1111/nph.15656 30632169

[32] Kramer K (1995). Phenotypic plasticity of the phenology of seven European tree species in relation to climatic warming. Plant Cell Environ.

[33] Vitasse Y, Bresson CC, Kremer A, Michalet R, Delzon S (2010). Quantifying phenological plasticity to temperature in two temperate tree species. Funct Ecol.

[34] Tansey CJ, Hadfield JD, Phillimore AB (2017). Estimating the ability of plants to plastically track temperature-mediated shifts in the spring phenological optimum. Glob Change Biol..

[35] Callaway RM, Pennings SC, Richards CL (2003). Phenotypic plasticity and interactions among plants. Ecology.

[36] Friend AD, Woodward FI, Switsur VR (1989). Field measurements of photosynthesis, stomatal conductance, leaf nitrogen and δ^13^C along altitudinal gradients in Scotland. Funct Ecol.

[37] Oleksyn J, Modrzýnski J, Tjoelker MG, Z ytkowiak R, Reich PB, Karolewski P (1998). Growth and physiology of Picea abies populations from elevational transects: common garden evidence for altitudinal ecotypes and cold adaptation. Funct Ecol.

[38] Soolanayakanahally RY, Guy RD, Silim SN, Drewes EC, Schroeder WR (2009). Enhanced assimilation rate and water use efficiency with latitude through increased photosynthetic capacity and internal conductance in balsam poplar (*Populus balsamifera* L.). Plant Cell Environ.

[39] Sharkey TD, Bernacchi CJ, Farquhar GD, Singsaas EL (2007). Fitting photosynthetic carbon dioxide response curves for C_3_ leaves. Plant Cell Environ.

[40] Cernusak LA, Ubierna N, Winter K, Holtum JA, Marshall JD, Farquhar GD (2013). Environmental and physiological determinants of carbon isotope discrimination in terrestrial plants. New Phytol.

[41] Farquhar GD, Ehleringer JR, Hubick KT (1989). Carbon isotope discrimination and photosynthesis. Annu Rev Plant Physiol Plant Mol Biol.

[42] Beaman JE, White CR, Seebacher F (2016). Evolution of plasticity: mechanistic link between development and reversible acclimation. Trends Ecol Evol.

[43] Sándor R, Picon-Cochard C, Martin R, Louault F, Klumpp K, Borras D, Bellocchi G (2018). Plant acclimation to temperature: developments in the pasture simulation model. Field Crops Res.

[44] Pigliucci M (2005). Evolution of phenotypic plasticity: where are we going now?. Trends Ecol Evol.

[45] Via S, Lande R (1985). Genotype-environment interaction and the evolution of phenotypic plasticity. Evolution.

[46] Van Tienderen PH (1991). Evolution of generalists and specialist in spatially heterogeneous environments. Evolution.

[47] Hendry AP (2016). Key questions on the role of phenotypic plasticity in eco-evolutionary dynamics. J Hered.

[48] Xie C-Y, Ying CC, Yanchuk AD, Holowachuk DL (2009). Ecotypic mode of regional differentiation caused by restricted gene migration: a case in black cottonwood (*Populus trichocarpa*) along the Pacific northwest coast. Can J For Res.

[49] Crispo E, DiBattista JD, Correa C, Thibert-Plante X, McKellar AE, Schwartz AK, Berner D, De León LF, Hendry AP (2010). The evolution of phenotypic plasticity in response to anthropogenic disturbance. Evol Ecol Res.

[50] Morrissey MB, Hadfield JD (2012). Directional selection in temporally replicated studies is remarkably consistent. Evolution.

[51] Siepielski AM, DiBattista JD, Carlson SM (2009). It’s about time: the temporal dynamics of phenotypic selection in the wild. Ecol Lett.

[52] Porcher E, Giraud T, Goldringer I, Lavigne C (2004). Experimental demonstration of a casual relationship between heterogeneity of selection and genetic differentiation in quantitative traits. Evolution.

[53] Franks SJ (2011). Plasticity and evolution in drought avoidance and escape in the annual plant *Brassica rapa*. New Phytol.

[54] Heschel MS, Riginos C (2005). Mechanisms of selection for drought stress tolerance and avoidance in *Impatiens capensis* (Balsaminaceae). Am J Bot.

[55] Franks SJ, Sim S, Weis AE (2007). Rapid evolution of flowering time by an annual plant in response to a climate fluctuation. Proc Natl Acad Sci U S A.

[56] Kingsolver JG, Diamond SE, Siepielski AM, Carlson SM (2012). Synthetic analyses of phenotypic selection in natural populations: lessons, limitations and future directions. Evol Ecol.

[57] Bell G (2010). Fluctuating selection: the perpetual renewal of adaptation in variable environments. Philos Trans R Soc B Biol Sci.

[58] Phillips PC, Arnold SJ (1989). Visualizing multivariate selection. Evolution.

[59] McGlothlin JW (2010). Combining selective episodes to estimate lifetime nonlinear selection. Evolution.

[60] Arnold SJ, Burger R, Hohenlohe PA, Ajie BC, Jones AG (2008). Understanding the evolution and stability of the G-matrix. Evolution.

[61] Conover DO, Schultz ET (1995). Phenotypic similarity and the evolutionary significance of countergradient variation. Trends Ecol Evol.

[62] Evans M, Aubriot X, Hearn D, Lanciaux M, Lavergne S, Cruaud C, Lowry PP, Haevermans T (2014). Insights on the evolution of plant succulence from a remarkable radiation in Madagascar (*Euphorbia*). Syst Biol.

[63] Zanne AE, Tank DC, Cornwell WK, Eastman JM, Smith SA, FitzJohn RG, McGlinn DJ, O’Meara BC, Moles AT, Reich PB (2013). Three keys to the radiation of angiosperms into freezing environments. Nature.

[64] Liu Y, El-Kassaby YA (2018). Evapotranspiration and favorable growing degree-days are key to tree height growth and ecosystem functioning: meta-analyses of Pacific northwest historical data. Scientifc Reports.

[65] St Clair JB, Howe GT (2007). Genetic maladaptation of coastal Douglas-fir seedlings to future climates. Glob Change Biol.

[66] Pickles BJ, Twieg BD, O'Neill GA, Mohn WW, Simard SW (2015). Local adaptation in migrated interior Douglas-fir seedlings is mediated by ectomycorrhizas and other soil factors. New Phytol.

[67] McKown AD, Guy RD, Azam MS, Drewes EC, Quamme LK (2013). Seasonality and phenology alter functional leaf traits. Oecologia.

[68] Geraldes A, Farzaneh N, Grassa CJ, McKown AD, Guy RD, Mansfield SD, Douglas CJ, Cronk QCB (2014). Landscape genomics of *Populus trichocarpa*: the role of hybridization, limited gene flow, and natural selection in shaping patterns of population structure. Evolution.

[69] McKown AD, Guy RD, Klápště J, Geraldes A, Friedmann M, Cronk QCB, El-Kassaby YA, Mansfield SD, Douglas CJ (2014). Geographical and environmental gradients shape phenotypic trait variation and genetic structure in *Populus trichocarpa*. New Phytol.

[70] R Core Team: R: a language and environment for statistical computing. In. Vienna, Austria: R Foundation for Statistical Computing; 2018.

[71] Holm S (1979). A simple sequentially rejective multiple test procedure. Scand J Stat.

[72] Kuznetsova A, Brockhoff PB (2017). Christensen RHB: lmerTest package: tests in linear mixed effects models. J Stat Softw.

[73] Bates D, Machler M, Bolker BM, Walker SC (2015). Fitting linear mixed effects models using lme4. J Stat Softw.

[74] Kawecki TJ, Ebert D (2004). Conceptual issues in local adaptation. Ecol Lett.

[75] Lenth RV (2016). Least-squares means: the R package lsmeans. J Stat Softw.

[76] McKay JK, Latta RG (2002). Adaptive population divergence: markers, QTL and traits. Trends Ecol Evol.

[77] Leinonen T, McCairns RJ, O'Hara RB, Merilä J (2013). *Q*_ST_-*F*_ST_ comparisons: evolutionary and ecological insights from genomic heterogeneity. Nat Rev Genet.

[78] Spitze K (1993). Population structure in Daphnia obtusa: quantitative genetic and allozymic variation. Genetics.

[79] Hadfield JD (2010). MCMC methods for multi-response generalized linear mixed models: the MCMCglmm R package. J Stat Softw.

[80] Merilä J, Crnokrak P (2001). Comparison of genetic differentiation at marker loci and quantitative traits. J Evol Biol.

[81] Scheiner SM, Lyman RF (1989). The genetics of phenotypic plasticity I. Heritability. J Evol Biol.

[82] Lande R, Arnold SJ (1983). The measurement of selection on correlated characters. Evolution.

[83] Conner JK, Hartl DL (2004). A primer of ecological genetics.

[84] De Lisle SP, Svensson EI (2017). On the standardization of fitness and traits in comparative studies of phenotypic selection. Evolution.

[85] Stinchcombe JR, Agrawal AF, Hohenlohe PA, Arnold SJ, Blows MW (2008). Estimating nonlinear selection gradients using quadratic regression coefficients: double or nothing?. Evolution.

[86] Pagel M, Meade A: BayesTraits. Computer program and documentation available at http://www.evolution.rdg.ac.uk/BayesTraits.html. ; 2007.

[87] Liu Y, El-Kassaby Y: Phenotypic plasticity of natural *Populus trichocarpa* populations in response to temporally environmental change in a common garden. Dryad Dataset. 2019; doi:org/10.5061/dryad.02v6wwq00. 10.1186/s12862-019-1553-6 PMC6933736 31878866

