# Correction to: Phenotypic plasticity of natural *Populus trichocarpa* populations in response to temporally environmental change in a common garden

**DOI:** 10.1186/s12862-021-01884-9

**Published:** 2021-08-09

**Authors:** Yang Liu, Yousry A. El-Kassaby

**Affiliations:** Department of Forest and Conservation Sciences, The University of British Columbia, 2424 Main Mall, Vancouver, British Columbia V6T 1Z4 Canada

## Correction to: BMC Evol Biol (2019) 19:231 https://doi.org/10.1186/s12862-019-1553-6

Following the publication of the original article [1], the authors have flagged that the link to the original data provided in the article is incorrect.

Information about the original data used in that article is viewable at: https://dataverse.scholarsportal.info/dataset.xhtml?persistentId=doi:10.5683/SP2/OAXHHZ
