# Supplementary material for: Phenotypic plasticity of natural Populus trichocarpa populations in response to temporally environmental change in a common garden
Source: BMC Evol Biol. 2019 Dec 26;19:231. doi: 10.1186/s12862-019-1553-6 (PMC6933736; doi:10.1186/s12862-019-1553-6)
Supplement: Supplementary file 1 — Additional file 1. Table S1. Information about the study populations. Table S2. Pairwise Spearman’s rank correlations for the trait genetic relationship among the 29 populations calculated using population-level ordinary least-squares mean trait values. Table S3. Statistics from REML-linear mixed models of ecophysiology traits for the comparison of trait responses across 29 Populations in two years (i.e. temporal Environments) and Year × Population interactions. Table S4. Linear phenotypic selection analysis (selection differentials) of the 18 traits over two years. Table S5. Non-linear (quadratic) selection differentials of the 18 traits over two years. Table S6. Test for heterogeneity of directional selection on the 18 traits between years. Table S7. Linear phenotypic selection gradients of the 18 traits over two years. Figure S1. 29 natural Populus trichocarpa populations marked on the map with this species’ geographic distribution range shaded in grey. Figure S2. Climate of the common garden over the trait measurement periods 2008-2010. Figure S3. Best linear unbiased predictions (BLUPs) for each genotype by trait. Figure S4. Estimated phenology and biomass trait values for each genotype compared by year. Figure S5. Population and temporal environment affects ecophysiology traits independently. Figure S6. Population and temporal environment affects ecophysiology traits through their interactions. Figure S7. Correlation between standardized traits (Z-scores) and relative fitness (scaled height gain over single years). Figure S8. Fitness (height gain) and plasticity (reaction norm) relationship for the study traits. Figure S9. Visualization of linear selection differentials for each of the 18 traits. Figure S10. Visualization of quadratic selection differentials for each of the 18 traits. Figure S11. Present-day P. trichocarpa suitability scores across its distribution range. Figure S12. Individual phylogeny based on individual Euclidean genetic distance. Note S1. [file 12862_2019_1553_MOESM1_ESM.pdf]

SUPPLEMENTAL INFORMATION

Phenotypic plasticity of natural *Populus trichocarpa* populations in response to temporally environmental change in a common garden

Yang LIU, Yousry A. EL-KASSABY

Department of Forest and Conservation Sciences, The University of British Columbia

2424 Main Mall, Vancouver, British Columbia V6T 1Z4 Canada

Supplementary Tables

Table S1 Information about the study populations

Popltn number	Popltn name	Latitude range (°N)	Longitude range (°W)	Altitude range (masl)	Deme group*	$N_{provenances}$	$N_{genotypes}$
1	Alsek	59.43-59.62	137.83-137.92	34-107	northern	2	4
3	Taku	58.7-58.93	133.4-133.18	49-122	northern	3	5
4	Upper Stikine	57.95	129.67	732	northern	2	2
5	Lower Stikine	57.33-57.65	131.57-131.78	49-85	northern	3	4
6	Iskut	56.7-56.93	130.33-131.15	73-317	northern	3	8
7	Bell-Irving	56.73-56.85	129.62-129.82	579-677	northern	3	7
8	Nass	55.05-55.72	128.78-129.5	24-183	northern	4	9
9	Kispiox	55.77	128.53	518	northern	1	1
10	Skeena	54.22-55.22	127.67-129.53	21-305	northern	16	38
11	Bulkley	54.45-55.52	126.8-127.5	311-579	northern	5	11
12	Kitimat	54.05-54.25	128.45-128.68	18-335	northern	4	10
13	Stuart	54.6	124.77	686	northern	1	1
14	Nechako	54.03	124.92	701	northern	2	2
15	McGregor	53.92-54.18	122-122.6	564-640	northern	3	14
16	Quesnel	52.72-53.4	121.17-122.87	442-823	northern	7	27
17	Dean	52.77-52.83	126.62-126.95	27-213	southern	4	11
18	Burke	52.38-52.42	126.17-126.6	135-152	southern	2	10
19	Chuckwall	51.62-51.77	126.58-127.32	67-122	southern	4	17
20	Klinaklini	50.98-51.73	125.5-126.23	0-427	southern	7	26
21	Homathko	50.82-51.28	124.48-124.95	37-239	southern	6	22
22	Philips	50.6-50.68	125.25-125.32	5-58	southern	2	10
23	Toba	50.52-50.57	124.08-124.23	67-73	southern	2	7
24	Jervis	50.22-50.25	123.93-124.02	61-323	southern	6	19
25	Squamish	49.87-50.25	123.23-123.58	61-305	southern	4	9
26	Lillooet	49.77-50.62	122.22-123.38	64-579	southern	8	24
27	Fraser	49.08-50	121.4-122.58	8-549	southern	15	41
28	Vancouver Island	49.07-50.28	123.87-126.05	20-213	southern	11	37
29	Columbia	45.58-46.08	122-123.92	100-900	Oregon	4	10
30	Willamette	44-45.57	122.92-123.33	100-300	Oregon	5	17

Popltn: population. No popltn #2 in this study.

* Three deme groups defined through classification of population structure (Geraldes *et al.* 2014). Oregon is the southernmost deme in our study.

N is the number of individuals in each population classified by provenance and genotype. In total, 403 genotypes from 139 provenances were used.

Table S2 Pairwise Spearman's rank correlations for the trait genetic relationship among the 29 populations calculated using population-level ordinary least-squares mean trait values

	Bud set	Post-bud set period	Growth period	Canopy duration	Leaf drop	Active growth rate	Height : diameter
Bud set		***	***	***	***	***	
Post-bud set period	**-0.99**		***	***	***	***	
Growth period	**0.98**	**-0.97**		***	***	***	
Canopy duration	**0.94**	**-0.92**	**0.98**		***	***	
Leaf drop	**0.98**	**-0.95**	**0.97**	**0.94**		***	
Active growth rate	**0.82**	**-0.80**	**0.81**	**0.81**	**0.85**		
Height : diameter	0.33	-0.30	0.28	0.28	0.29	0.22	

	A_{max}	$A_{max/mass}$	C : N	$\delta^{15}N$	Δ_{leaf}	g_s	LMA_{summer}	N_{area}	N_{mass}	NUE	WUE
A_{max}		***	*			***		***	**		
$A_{max/mass}$	**0.89**		**			**		**	***		
C : N	**-0.63**	**-0.79**						***	***		
$\delta^{15}N$	0.38	0.41	-0.59								
Δ_{leaf}	0.27	0.16	0.14	0.48		*				**	***
g_s	**0.83**	**0.68**	-0.35	0.54	**0.65**					**	***
LMA_{summer}	-0.11	-0.30	0.38	-0.16	0.04	-0.13					
N_{area}	**0.78**	**0.70**	**-0.78**	0.29	-0.21	0.46	-0.09		***		
N_{mass}	**0.67**	**0.80**	**-0.99**	0.43	-0.24	0.35	-0.37	**0.83**			
NUE	0.61	0.58	-0.03	0.23	**0.71**	**0.72**	-0.05	0.06	0.05		*
WUE	-0.58	-0.42	0.23	-0.60	**-0.84**	**-0.87**	-0.02	-0.22	-0.14	**-0.63**	

Significance: * $P < 0.05$, ** $P < 0.01$, *** $P < 0.001$

Spearman's rank correlation coefficient ρ is given for each pair of traits in the lower diagonal of the matrix table. Population ordinary least squares mean trait values were generated from the variance partitioning (OLS model) described in Table 2 (for phenology and biomass traits) and Table S3 (for ecophysiology traits).

Table S3 Statistics from REML-linear mixed models of ecophysiology traits for the comparison of trait responses across 29 Populations in two years (i.e. temporal Environments) and Year × Population interactions

		A_{max}			$A_{max/mass}$			C : N			Δ_{leaf}			$\delta^{15}N$			g_s		
	N DF	D DF	*F*/ χ^2	*P*	D DF	*F*/ χ^2	*P*	D DF	*F*/ χ^2	*P*	D DF	*F*/ χ^2	*P*	D DF	*F*/ χ^2	*P*	D DF	*F*/ χ^2	*P*
Popltn	25	422	5.01	**$<10^{-4}$**	526	5.69	**$<10^{-4}$**	423	5.37	**$<10^{-4}$**	443	5.86	**$<10^{-4}$**	431	2.82	**$<10^{-4}$**	435	9.06	**$<10^{-4}$**
Year	1	407	1.09	1	526	3.84	0.355	438	28.75	**$<10^{-4}$**	361	0.35	1	388	8.54	**0.033**	369	77.08	**$<10^{-4}$**
Popltn × Year	25	408	2.05	**0.025**	526	1.15	1	430	0.89	1	360	0.90	1	388	1.67	0.241	358	1.08	1
Genetics	1	NA	1.07	1	NA	0	1	NA	0.04	1	NA	16.77	**0.0005**	NA	4.33	0.337	NA	15.46	**0.001**
Model fit statistics	R^2		0.356			0.291			0.310			0.546			0.383			0.668	
	AIC		2836.19			-1723.09			2902.85			1727.88			1377.72			-1129.85	
	REML		2728.19			-1831.09			2796.85			1619.88			1269.72			-1235.85	

		LMA_{summer}			N_{area}			N_{mass}			NUE			WUE		
	N DF	D DF	*F*/ χ^2	*P*	D DF	*F*/ χ^2	*P*	D DF	*F*/ χ^2	*P*	D DF	*F*/ χ^2	*P*	D DF	*F*/ χ^2	*P*
Popltn	25	474	2.46	**0.0004**	413	3.55	**$<10^{-4}$**	525	4.78	**$<10^{-4}$**	439	1.93	**0.01**	428	4.97	**$<10^{-4}$**
Year	1	516	157.79	**$<10^{-4}$**	413	1.18	1	525	6.19	0.105	433	0.06	1	406	58.05	**$<10^{-4}$**
Popltn × Year	25	429	1.18	1	415	1.50	0.53	525	1.45	0.597	433	0.89	1	407	1.88	0.069
Genetics	1	NA	1.57	1	NA	0.23	1	NA	0	1	NA	0.82	1	NA	1.864	1
Model fit statistics	R^2		0.489			0.242			0.276			0.198			0.447	
	AIC		-4039.42			-6914.05			-4340.91			2158.41			1184.11	
	REML		-4139.42			-7022.05			-4448.91			2050.41			1076.11	

We report on *F*-values for fixed effects and on χ^2 values for random effects (i.e. 'Genetics'). Significance of random effects was assessed using likelihood ratio tests. Significant *P*-values after sequential Bonferroni corrections (α = 0.05) are shown in bold. Denominator degrees of freedom (D DF) are calculated using the Satterthwaite's approximation as implemented in lmerTest of R package. Popltn: Population

Due to rank deficiency of fixed effect model matrix for LMA_{summer}, g_s and C : N, several columns were dropped and N DF reduced to 21, 24 and 24, respectively. Moreover, only singular fits were obtained for $A_{max/mass}$ and N_{mass}-based models.

Table S4 Linear phenotypic selection analysis (selection differentials) of the 18 traits over two years

Trait	Year	N DF	D DF	*F*	*P*
**Bud set**	2009	1	363	524.41	**0**
	2010	1	203	852.46	**0**
**Canopy duration**	2009	1	393	76.4	**0**
	2010	1	391	161.82	**0**
**Growth period**	2009	1	395	224.76	**0**
	2010	1	336	528.31	**0**
**Leaf drop**	2009	1	299	307.06	**0**
	2010	1	118	412.16	**0**
**Post-bud set period**	2009	1	393	178.29	**0**
	2010	1	399	469.31	**0**
**Height : diameter**	2009	1	388	38.14	**0**
	2010	1	398	0.19	**0**
**Active growth rate**	2009	1	379	1358.32	**0**
	2010	1	391	1872.82	**0**
A_{max}	2009	1	387	28.68	**0**
	2010	1	189	0.01	0.9402
$A_{max/mass}$	2009	1	387	12.92	**0.002**
	2010	1	188	3.96	0.0642
C : N	2009	1	382	3.07	0.1824
	2010	1	186	2.83	0.1824
$\boldsymbol{\Delta_{leaf}}$	2009	1	178	7.72	**0.006**
	2010	1	390	36.42	**0**
$\delta^{15}N$	2009	1	382	0.02	0.9004
	2010	1	186	1.01	0.634
$\boldsymbol{g_s}$	2009	1	389	40.22	**0**
	2010	1	189	10.9	**0.0036**
LMA_{summer}	2009	1	382	7.16	**0.0148**
	2010	1	315	2.53	0.1166
N_{area}	2009	1	383	0.16	1
	2010	1	187	0.06	1
N_{mass}	2009	1	383	4.81	0.0612
	2010	1	186	4.9	0.0612
NUE	2009	1	385	34.87	**0**
	2010	1	185	0.16	0.6924
**WUE**	2009	1	384	4.26	**0.0426**
	2010	1	189	13.49	**8.00E-04**

Denominator degrees of freedom (D DF) are calculated using the Satterthwaite's approximation as implemented in the R package lmerTest. *P*-values were calculated using permutation tests of trait values among individuals and were corrected for multiple comparisons (i.e. each trait tested separately in two years) using the sequential Bonferroni method. Significant traits (α = 0.05) and *P*-values are in bold.

Table S5 Non-linear (quadratic) selection differentials of the 18 traits over two years

Trait	Year	N DF	D DF	*F*	*P*
Bud set	**2009**	1	393	14.63	**0.0004**
	2010	1	400	2.06	0.1890
Canopy duration	2009	1	387	2.64	0.1604
	2010	1	396	3.59	0.1604
**Growth period**	**2009**	1	389	7.12	**0.0132**
	**2010**	1	396	25.91	**0.0000**
Leaf drop	2009	1	394	1.44	0.2626
	**2010**	1	400	83.88	**0.0000**
**Post-bud set period**	**2009**	1	390	12.51	**0.0018**
	**2010**	1	400	31.16	**0.0000**
**Height : diameter**	**2009**	1	389	15.23	**0.0024**
	**2010**	1	397	7.42	**0.0198**
Active growth rate	**2009**	1	392	9.06	**0.0080**
	2010	1	398	3.00	0.1198
A_{max}	2009	1	382	1.35	0.3042
	2010	1	187	5.02	0.1000
$A_{max/mass}$	**2009**	1	384	5.86	**0.0476**
	2010	1	185	1.11	0.3386
C : N	2009	1	380	1.79	0.3012
	2010	1	187	2.30	0.3012
Δ_{leaf}	2009	1	177	2.75	0.2008
	2010	1	387	2.46	0.2008
$\delta^{15}N$	2009	1	381	0.72	0.5396
	2010	1	183	1.23	0.5396
g_s	2009	1	384	3.68	0.0734
	**2010**	1	183	7.26	**0.0260**
LMA_{summer}	2009	1	381	0.84	0.3738
	2010	1	311	3.94	0.1024
N_{area}	2009	1	381	0.24	0.6342
	2010	1	186	1.41	0.5868
N_{mass}	2009	1	381	0.32	0.6050
	2010	1	185	1.91	0.4020
NUE	2009	1	381	0.11	0.7704
	2010	1	185	1.70	0.4424
WUE	2009	1	383	0.19	0.6886
	**2010**	1	186	7.73	**0.0088**

Denominator degrees of freedom (D DF) are calculated using the Satterthwaite's approximation as implemented in the R package lmerTest. *P*-values were calculated using permutation tests of trait values among individuals and were corrected for multiple comparisons (i.e. each trait tested separately in two years) using the sequential Bonferroni method. For each trait, significant year, trait (α = 0.05) and *P*-value are in bold.

Table S6 Test for heterogeneity of directional selection on the 18 traits between years

Trait	Term	N DF	D DF	*F*	*P*
**Bud set**	**Year**	1	789	33.76	**0**
	**β**	1	680	1286.11	**0**
	**Year × β**	1	790	57.44	**0**
**Canopy duration**	**Year**	1	788	16.76	**0**
	**β**	1	796	229.68	**0**
	**Year × β**	1	788	24.67	**0**
**Growth period**	**Year**	1	789	24.62	**0**
	**β**	1	789	699.53	**0**
	**Year × β**	1	789	53.14	**0**
**Leaf drop**	**Year**	1	789	22.85	**0**
	**β**	1	523	641.56	**0**
	**Year × β**	1	790	28.33	**0**
**Post-bud set period**	**Year**	1	789	23.38	**0**
	**β**	1	796	615.04	**0**
	**Year × β**	1	790	55.98	**0**
**Height : diameter**	**Year**	1	787	13.08	**0**
	**β**	1	791	13.05	**0**
	**Year × β**	1	788	10.25	**0.001**
**Active growth rate**	**Year**	1	788	65.47	**0**
	**β**	1	783	3133.61	**0**
	**Year × β**	1	789	118.39	**0**
**A_{max}**	**Year**	1	572	24.55	**0**
	**β**	1	577	5.81	**0.016**
	**Year × β**	1	574	7.85	**0.005**
$A_{max/mass}$	**Year**	1	571	24.61	**0**
	**β**	1	576	13.06	**0**
	Year × β	1	573	0	0.947
C : N	**Year**	1	570	23.24	0
	β	1	573	0.45	0.502
	**Year × β**	1	570	4.97	**0.026**
**Δ_{leaf}**	**Year**	1	572	4.34	**0.038**
	**β**	1	573	30.39	**0**
	**Year × β**	1	572	4.19	**0.041**
$\delta^{15}N$	**Year**	1	570	22.85	**0**
	β	1	572	0.97	0.324
	Year × β	1	570	0.68	0.411
g_s	**Year**	1	571	27.3	**0**
	**β**	1	578	40.78	**0**
	Year × β	1	571	0	0.977
LMA_{summer}	**Year**	1	698	88.48	**0**
	β	1	699	0.75	0.386
	**Year × β**	1	697	5.72	**0.017**
N_{area}	**Year**	1	571	23.37	**0**
	β	1	573	0.31	0.579
	Year × β	1	572	0.08	0.779
N_{mass}	**Year**	1	570	24.15	**0**
	β	1	573	0.78	0.376
	**Year × β**	1	570	8.01	**0.005**

**NUE**	**Year**	1	571	24.84	**0**
	**β**	1	573	10.27	**0.001**
	**Year × β**	1	573	5.84	**0.016**
**WUE**	**Year**	1	572	24.47	**0**
	**β**	1	575	20.76	**0**
	**Year × β**	1	573	6.12	**0.014**

Fixed effects are the terms in the second column from 18 separate REML-linear mixed models of relative fitness onto standardized trait values with year-by-trait interactions, after controlling the effect of unmeasured traits on fitness by including a random effect, 'Genetics'. β describes a test for nonzero mean directional selection throughout the experimental years, that is, *average selection*; Year × β describes a test for heterogeneous selection differentials between years, that is, *heterogeneous selection*.

Denominator degrees of freedom (D DF) are calculated using the Satterthwaite's approximation as implemented in the R package lmerTest. *P*-values were calculated using permutation tests of trait values among individuals and were corrected for multiple comparisons (i.e. each trait tested separately in two years) using the sequential Bonferroni method. Significant tests at α=0.05 were in bold.

Table S7 Linear phenotypic selection gradients of the 18 traits over two years

Trait	Year	β	N DF	D DF	*F*	*P*
Bud set	**2009**	**0.358**	1	165	26	**0**
	2010	-1.214	1	147	0.7	0.3482
Canopy duration	2009	0.410	1	161	0.74	1
	2010	-0.236	1	147	0.1	1
Growth period	2009	-0.426	1	161	0.49	1
	2010	0.651	1	147	0.22	1
Leaf drop	**2009**	**-0.197**	1	164	14.44	**0.0056**
	2010	0.496	1	147	0.58	0.357
Post-bud set period	2009	-0.044	1	161	0.02	0.9024
	**2010**	**-0.551**	1	147	11.73	**8.00E-04**
Height : diameter	2009	-0.002	1	165	0.04	0.8574
	2010	-0.008	1	147	2.31	0.2348
**Active growth rate**	**2009**	**0.243**	1	150	271.84	**0**
	**2010**	**0.368**	1	147	1974.38	**0**
A_{max}	2009	0.047	1	164	0.57	0.984
	2010	-0.009	1	147	0.04	0.984
$A_{max/mass}$	2009	0.049	1	166	0.8	0.8292
	2010	0.010	1	147	0.07	0.8292
C : N	2009	0.055	1	166	2.91	0.1888
	2010	0.029	1	147	2.16	0.1888
Δ_{leaf}	2009	0.001	1	163	0.01	0.9342
	2010	-0.010	1	147	2.33	0.2532
$\delta^{15}N$	2009	0.005	1	165	0.3	0.6736
	2010	0.006	1	147	1.03	0.6736
g_s	2009	-0.107	1	165	5.66	0.0724
	2010	0.020	1	147	0.62	0.4122
LMA_{summer}	2009	-0.062	1	165	1.63	0.2476
	2010	0.008	1	147	2.4	0.2476
N_{area}	2009	0.102	1	164	2.21	0.332
	2010	0.004	1	147	0.01	0.9266
N_{mass}	2009	-0.069	1	163	1.2	0.5808
	2010	0.016	1	147	0.21	0.6504
NUE	2009	-0.039	1	165	1.21	0.626
	2010	-0.012	1	147	0.14	0.7178
WUE	**2009**	**-0.082**	1	164	6.73	**0.0216**
	2010	0.003	1	147	0.04	0.8388

Denominator degrees of freedom (D DF) are calculated using the Satterthwaite's approximation as implemented in the R package lmerTest. *P*-values were calculated using permutation tests of trait values among individuals and were corrected for multiple comparisons (i.e. each trait tested separately in two years) using the sequential Bonferroni method.

Significant *F*-tests indicates nonzero selection gradients on a trait in a given year. The selection gradient describes direct selection on each trait after accounting for indirect selection on the others. It equals to the partial regression coefficient of relative fitness onto standardized trait values after including a random 'Genetics' intercept term, and for regression onto the other 17 traits.

Supplementary Figures

Figure S1 29 natural *Populus trichocarpa* populations marked on the map with this species' geographic distribution range shaded in grey

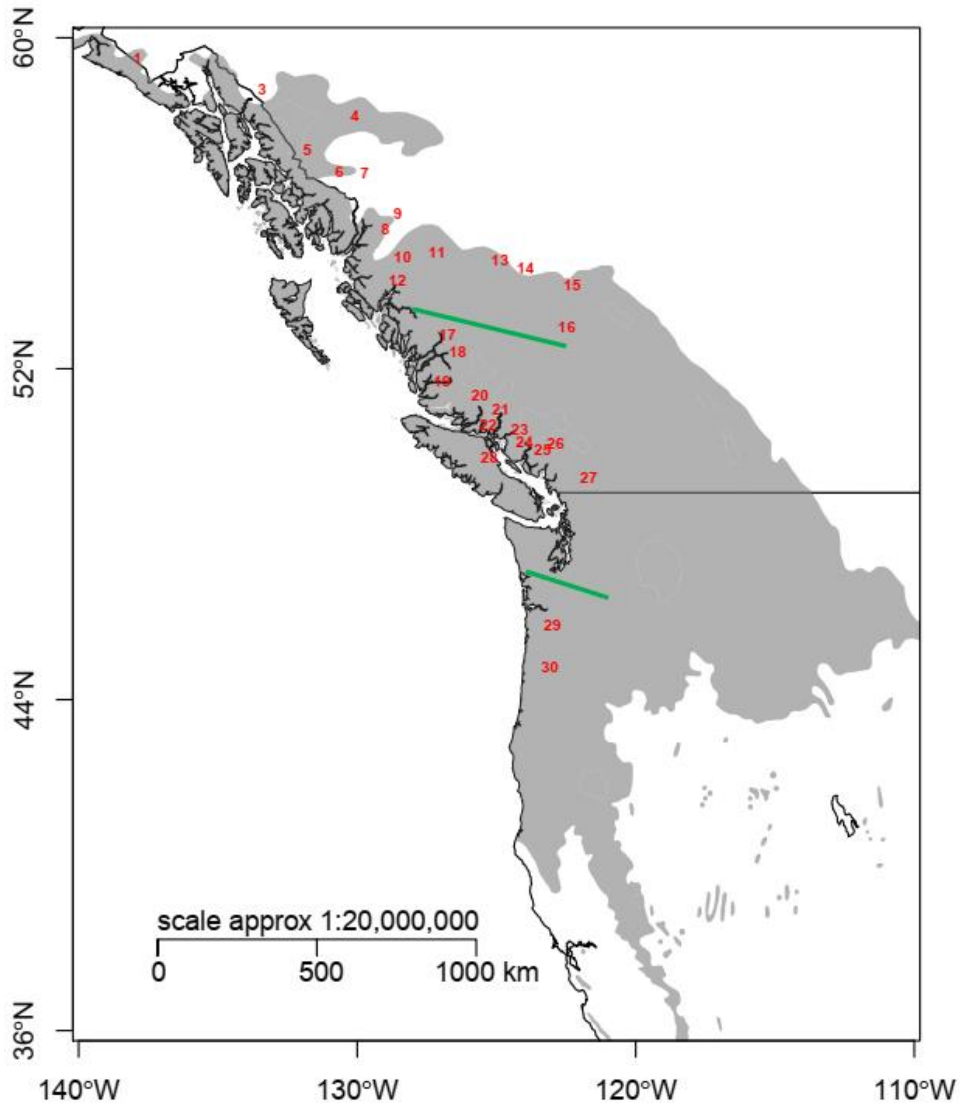

Two green transect lines divide the populations into three demes, that is, northern deme (including populations #1 - #16; no #2), southern deme (populations #17 - #28), and Oregon deme (southmost; populations #29 - #30).

Figure S2 Climate of the common garden over the trait measurement periods 2008-2010

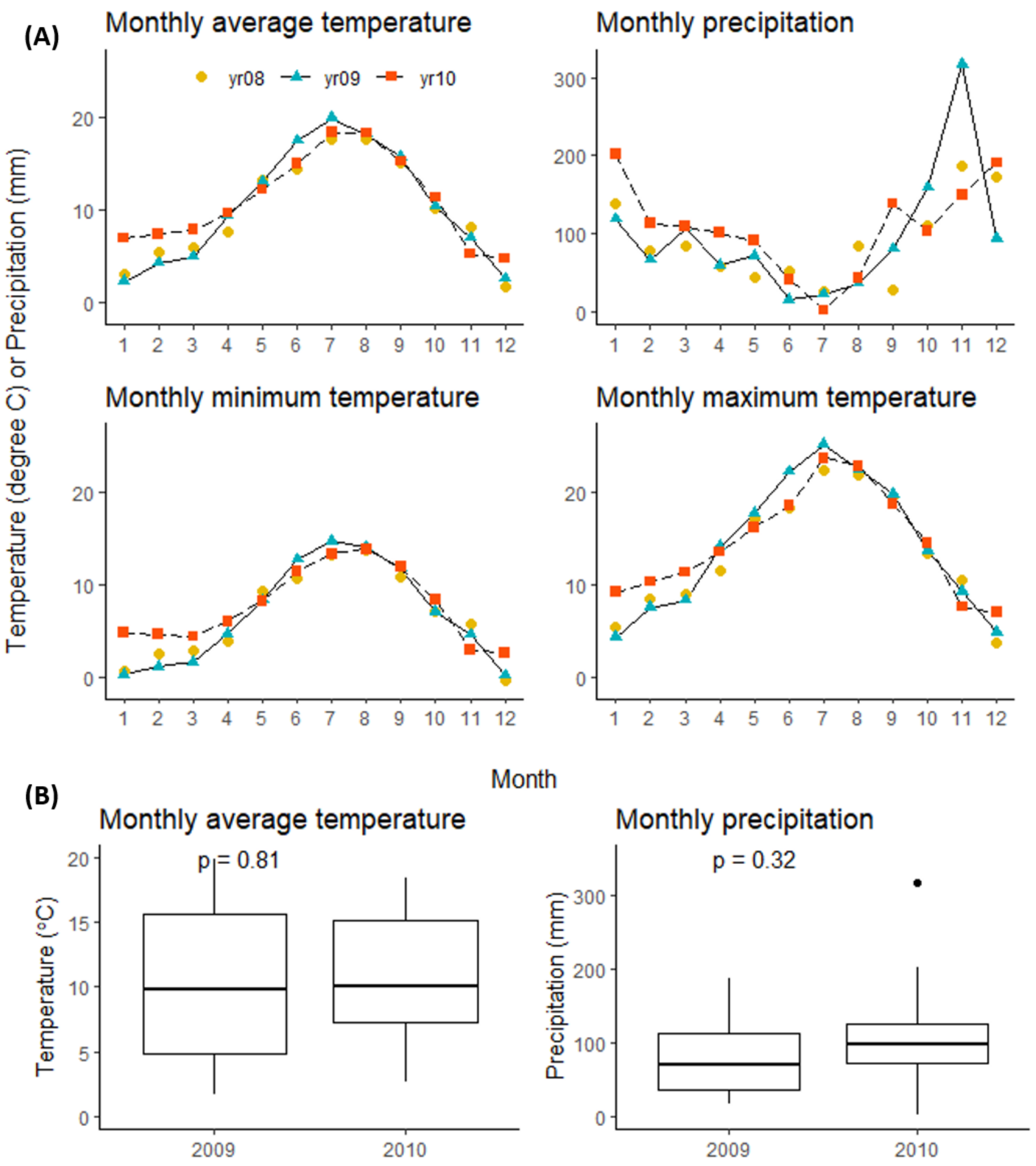

Note: yr08, yr09 and yr10 are the years of data collection. In 2008, only tree height was measured; all the traits (Table 1 in the main text for concise description) were measured in the following two years; thus, comparisons were made in 2009 vs. 2010. As trait measurements were performed in the spring for phenology and biomass traits and in May through August for ecophysiology traits (Note S1), we defined that climate for 2009 (2010) denotes temperature and precipitation in the period of September 2008 (2009) to August 2009 (2010), respectively. Paired *t*-test was used for the comparison between the two years in B.

Figure S3 Best linear unbiased predictions (BLUPs) for each genotype by trait

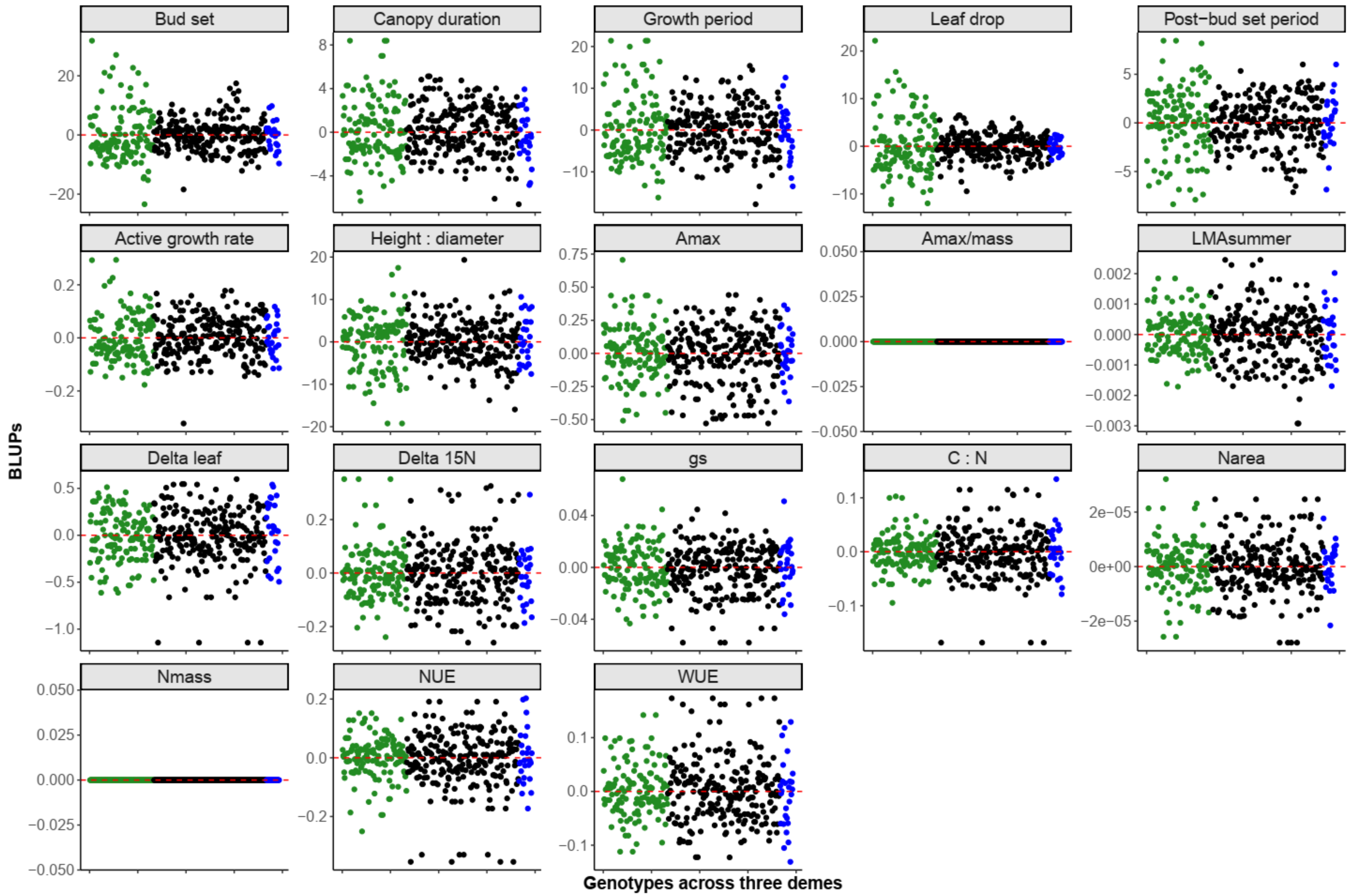

Only singular fits obtained for $A_{max/mass}$ and N_{mass}-based models. Green, black and blue dots denote populations in northern, southern and Oregon demes, respectively.

Figure S4 Estimated phenology and biomass trait values for each genotype compared by year

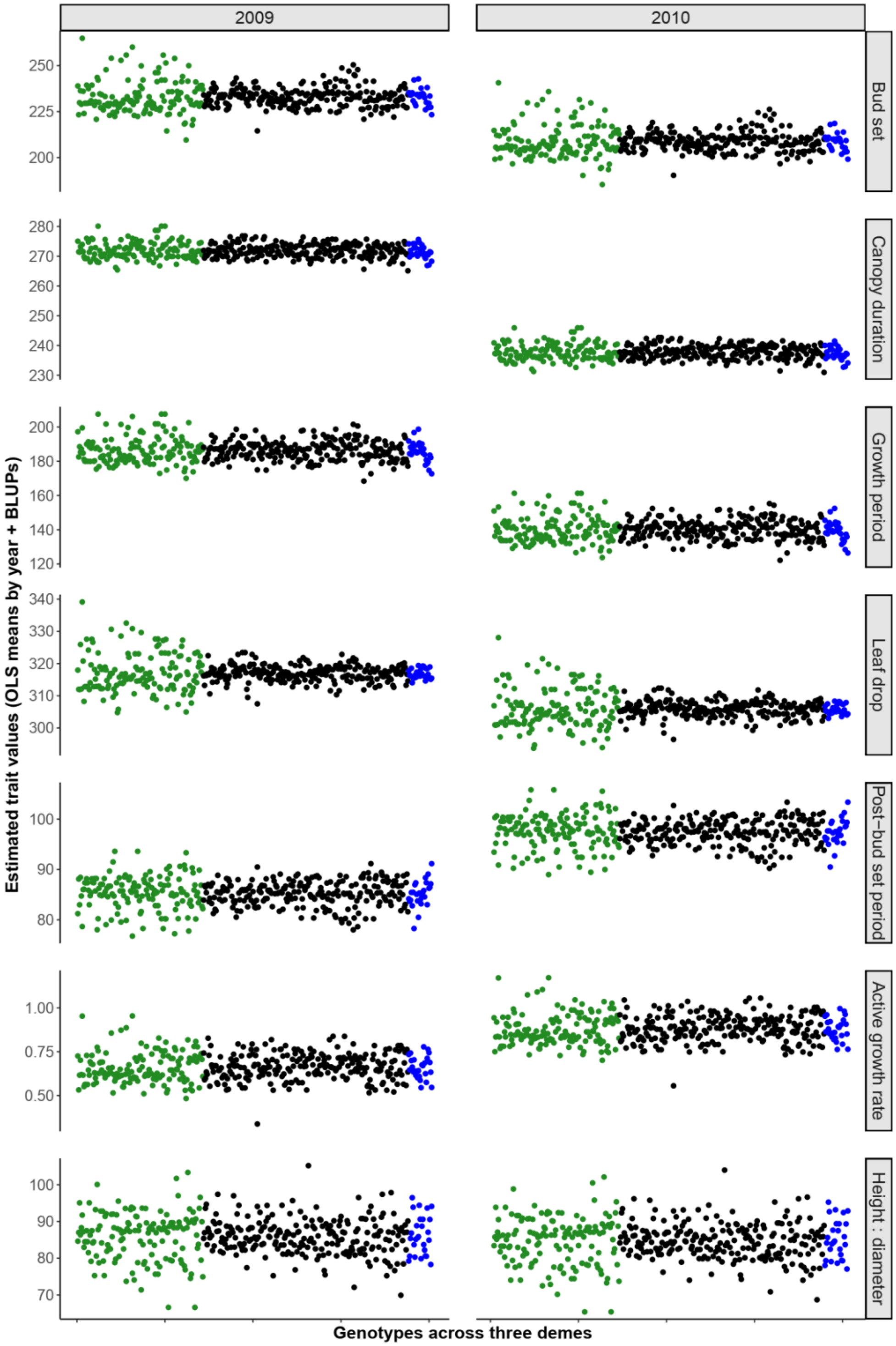

Best linear unbiased prediction (BLUP) is used in REML-linear mixed models for the estimation of random effects (**Zu** in Eqn.1 in Note S3). The random effects are the deviation from the "global average" (i.e. fixed effects). Together, estimated trait values in a given year = OLS mean trait values in that year + BLUPs for random effects from a REML-linear mixed model. Genotypes in three demes (northern, southern and Oregon) are distinguished in green, black and blue colors, respectively.

Figure S5 Population and temporal environment affects ecophysiology traits independently

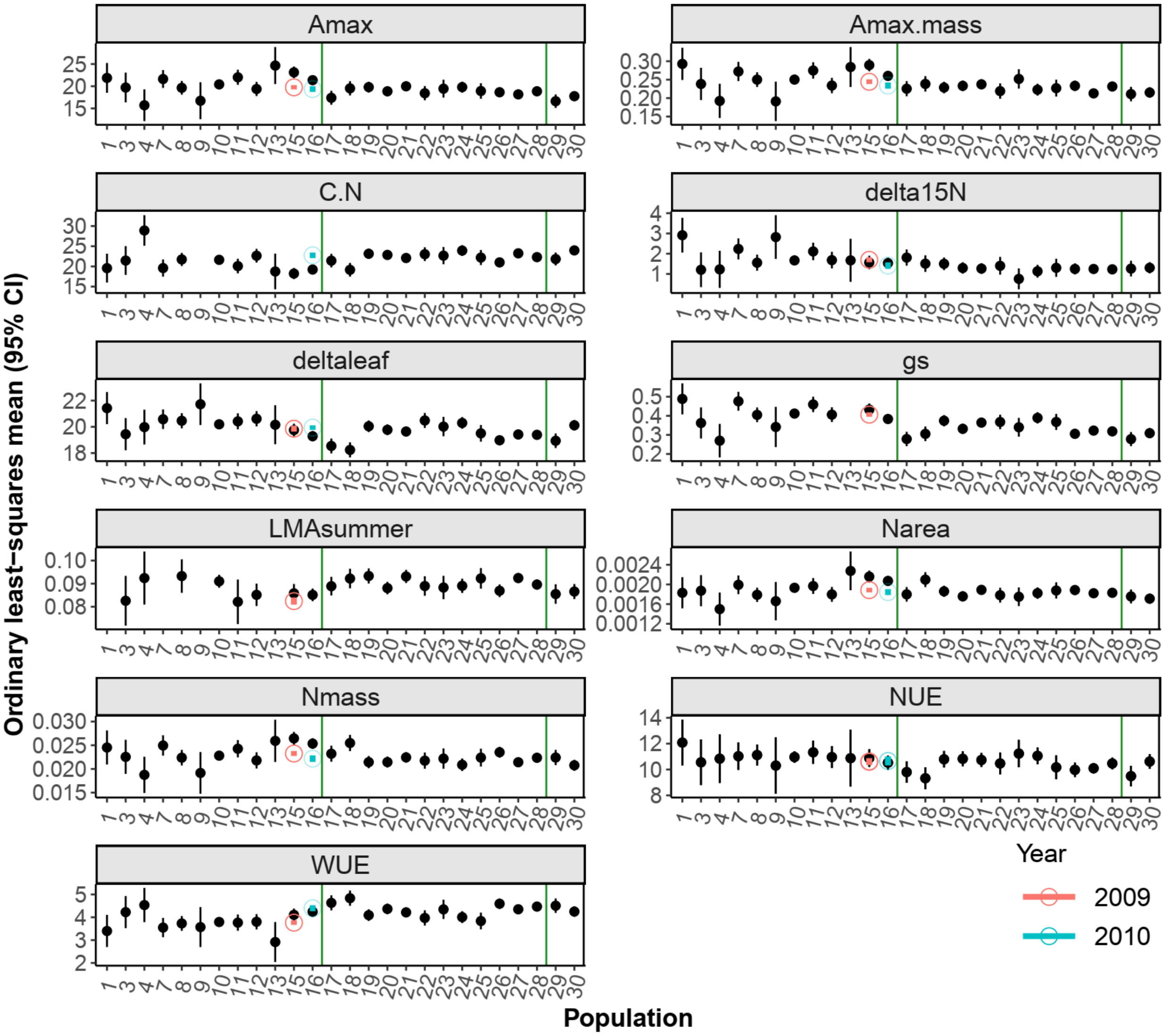

Both population and temporal environment affect the traits. The graph shows ordinary least-squares (OLS) mean trait values for each population (black filled circle) and for two years representing two temporal environments (red and blue empty-circles for 2009 and 2010, respectively) from a REML-linear mixed model (Table S3). Hence, black circles show the mean trait values for each population (averaged over years); colored circles show the mean trait value in each year (average across all populations). Note that the horizontal position of the colored circles is uninformative and they were just placed in the center of each panel. Error bars represent 95% CIs and vertical green lines delimit the populations into three demes, that is, northern, southern and Oregon (from left to right).

Figure S6 Population and temporal environment affects ecophysiology traits through their interactions

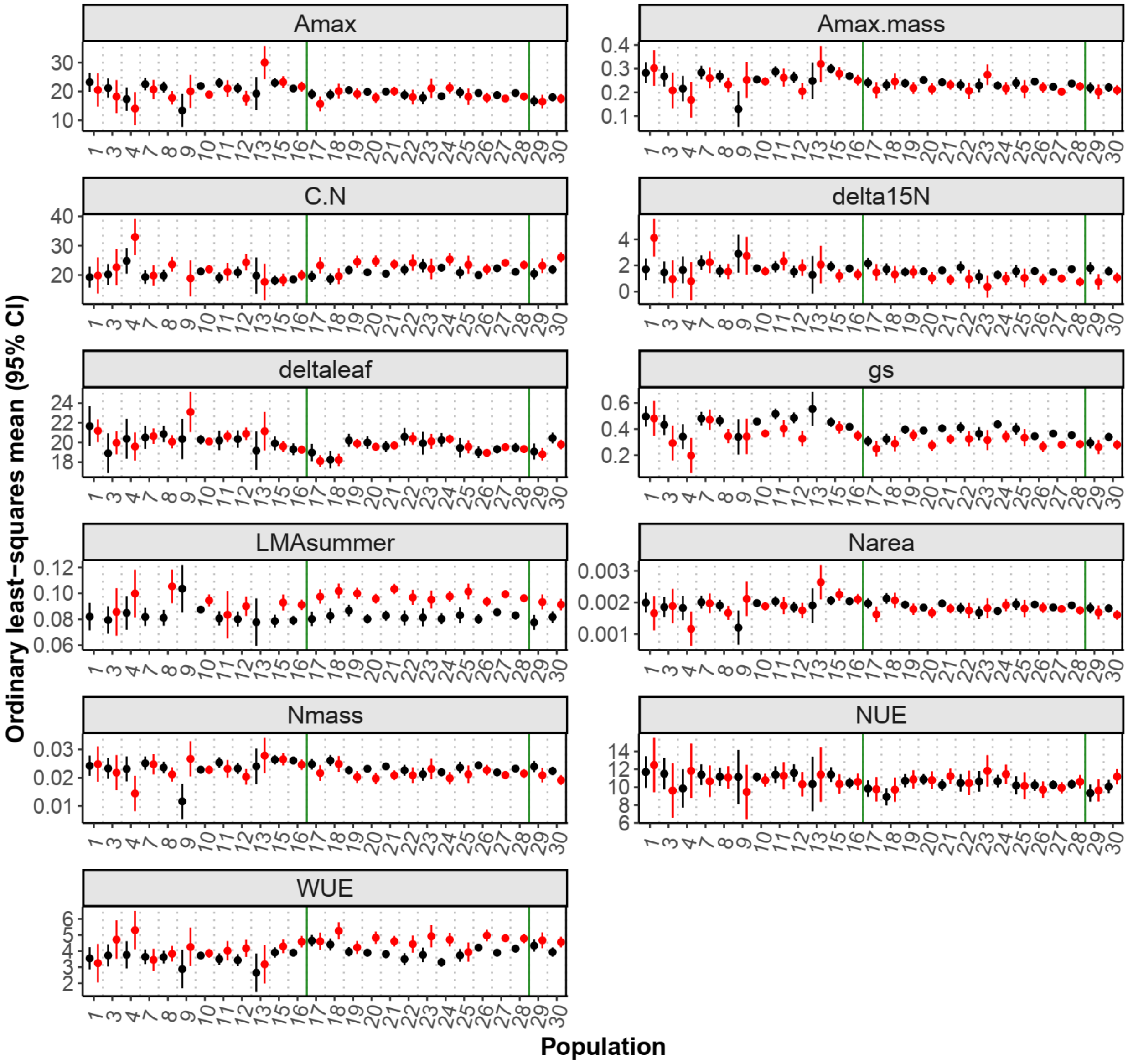

Phenotypical plasticity between years is variable in populations. Populations are separated by vertical dashed lines. The filled-circles are OLS mean trait values for each population over two years (black and red dots for 2009 and 2010, respectively) with 95% CIs.

Figure S7 Correlation between standardized traits (Z-scores) and relative fitness (scaled height gain over single years)

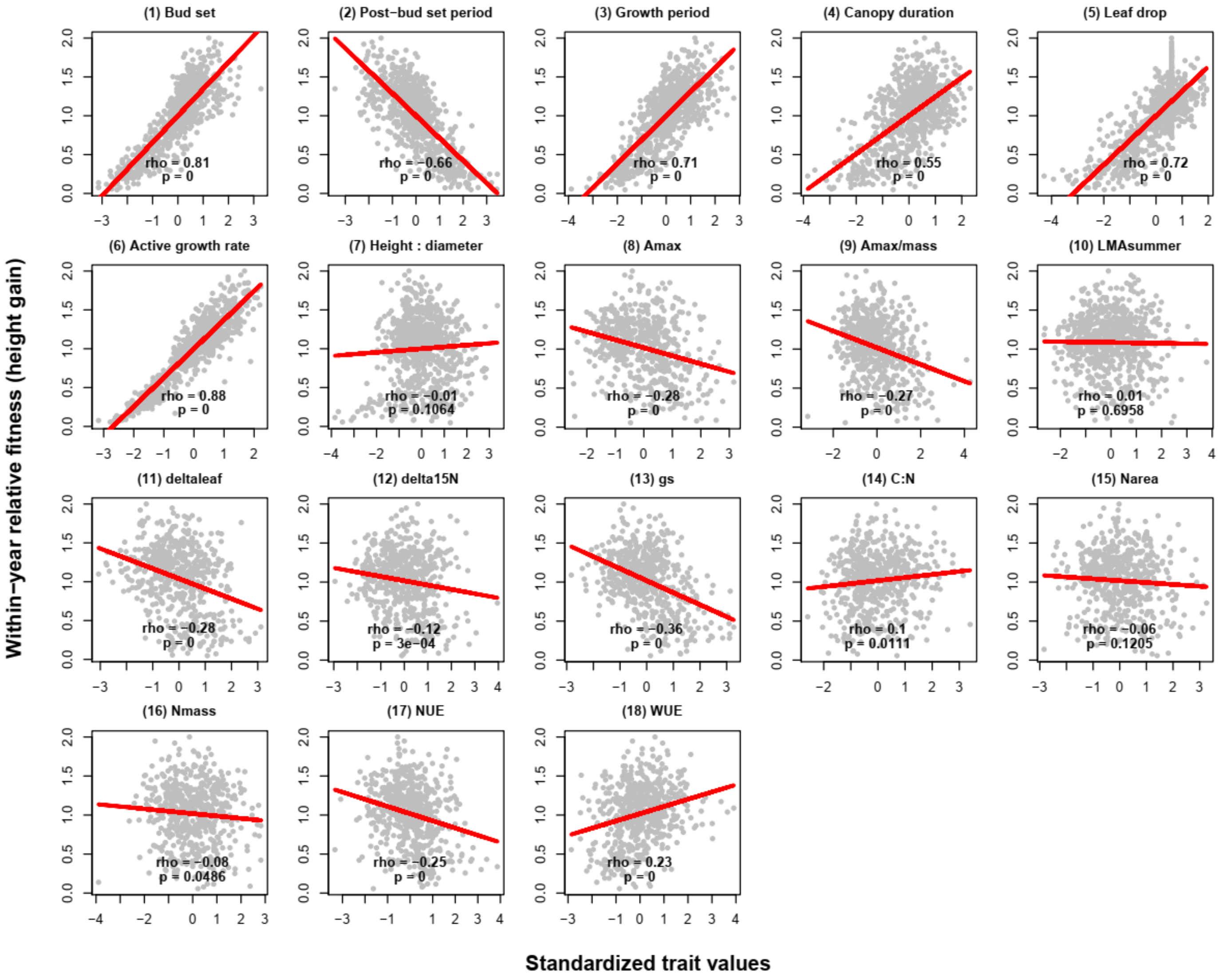

Pearson's rank correlation coefficients ρ and *P*-values were displayed in each panel. All *P*-values were adjusted using the sequential Bonferroni method.

Figure S8 Fitness (height gain) and plasticity (reaction norm) relationship for the study traits

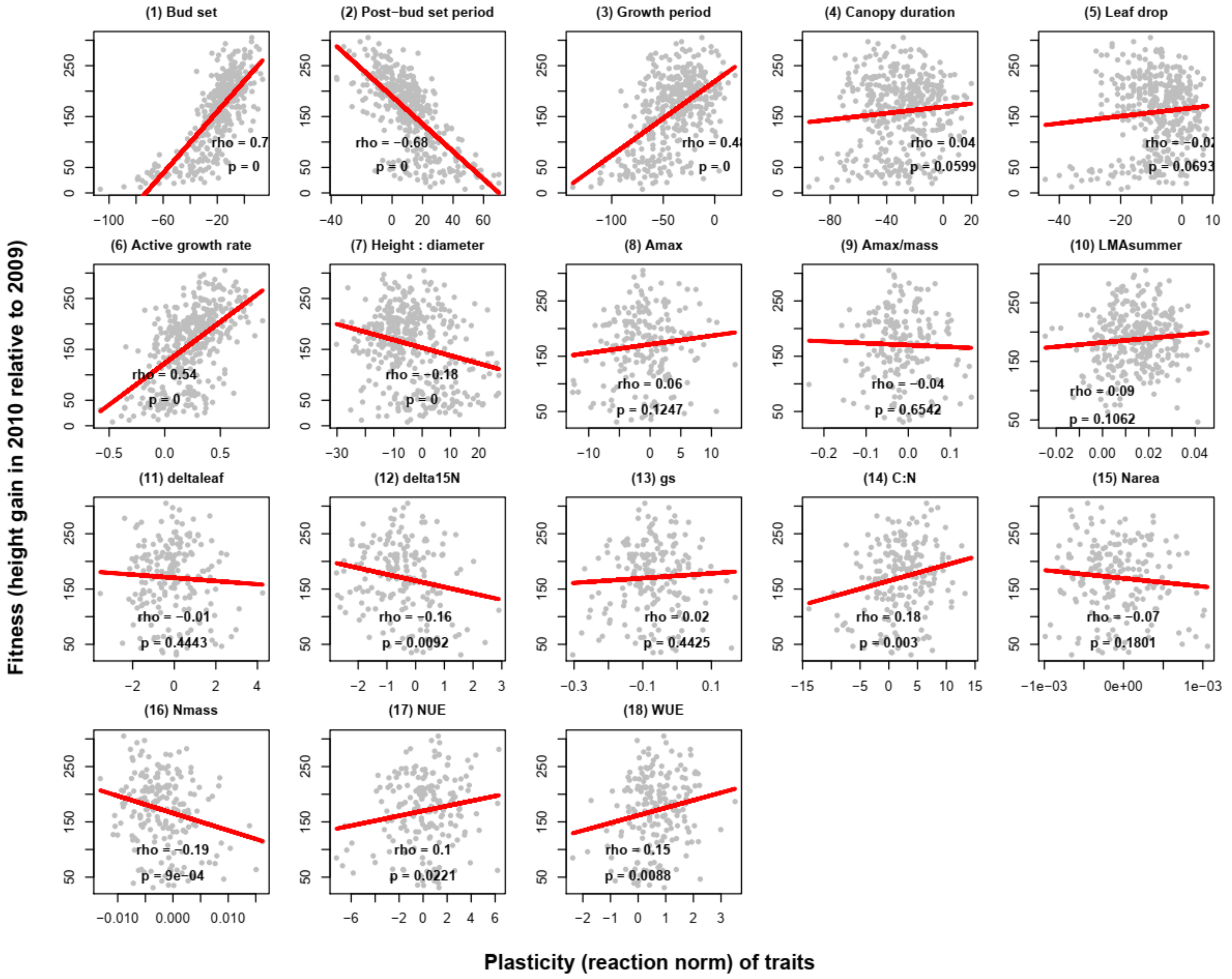

Pearson's rank correlation coefficients ρ and *P*-values were displayed in each panel. All *P*-values were adjusted using the sequential Bonferroni method.

Figure S9 Visualization of linear selection differentials for each of the 18 traits

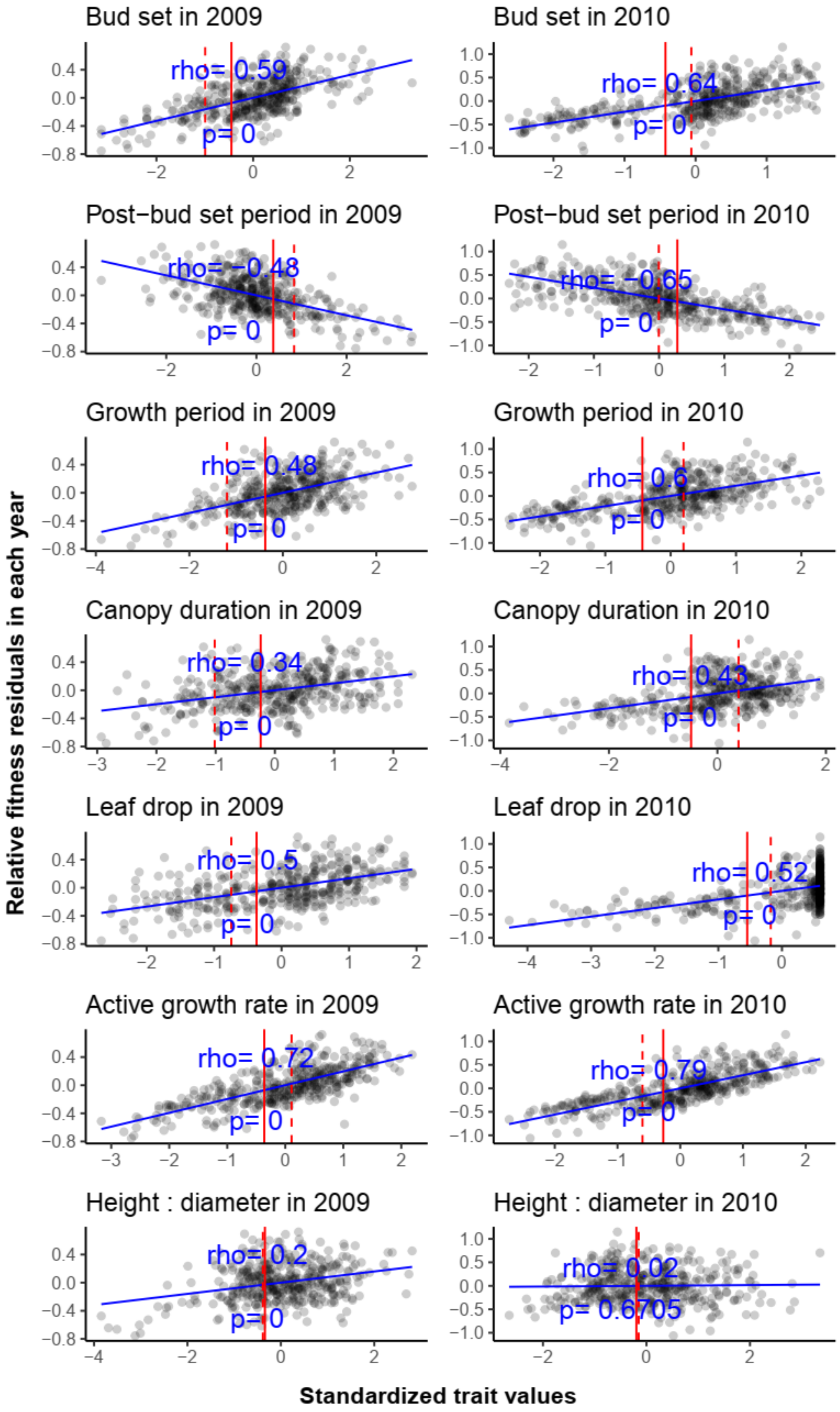

(N.B. next page for ecophysiology traits)

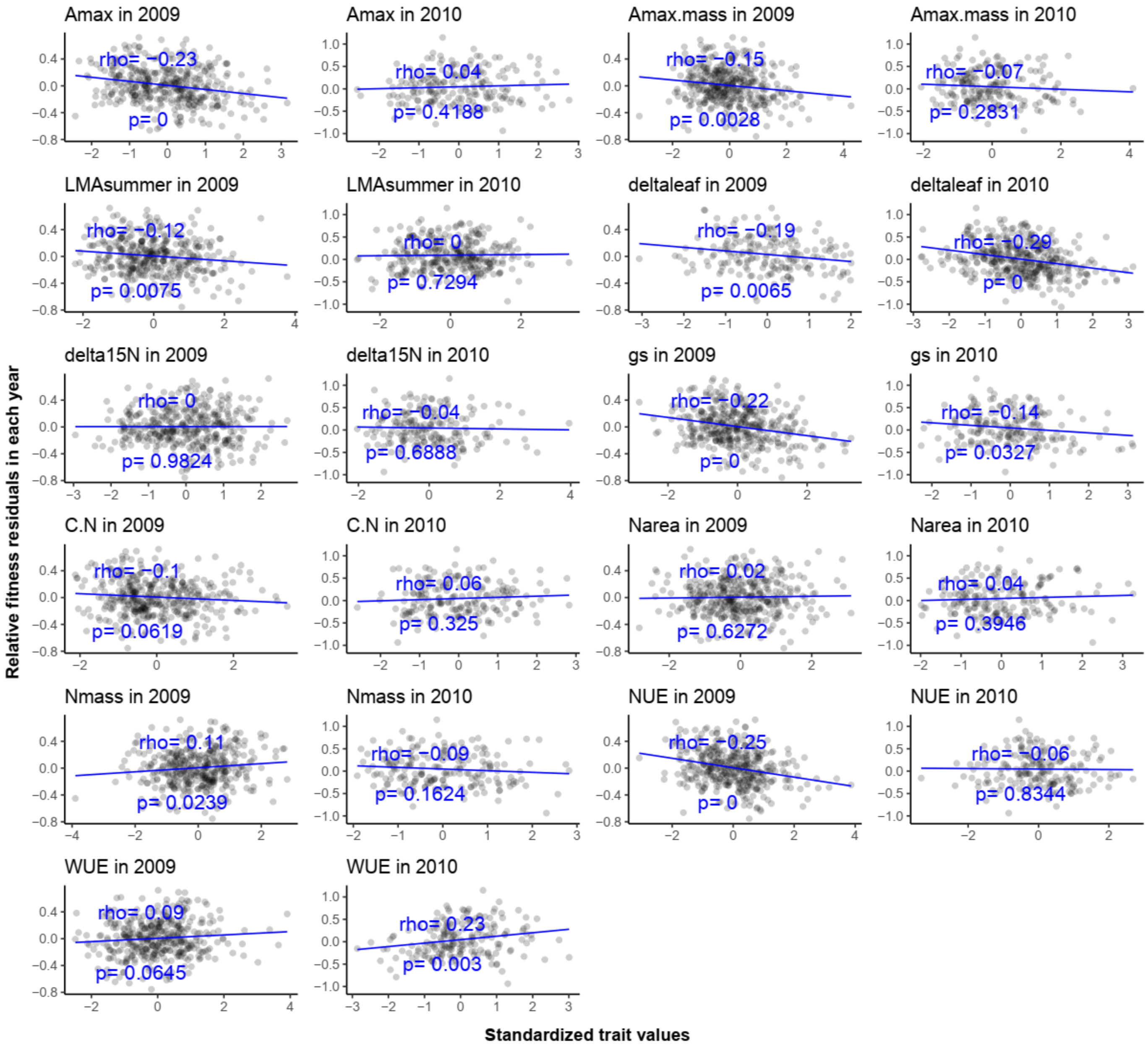

Note:
The average of phenology and biomass trait values (scaled OLS) across all genotypes in a given year and over the two years are delineated as dotted and solid vertical red lines, respectively (pg 18). Due to data missingness, these average values were not shown for ecophysiology traits.
Relative fitness residuals (details in Note S6) were used as a measure of fitness for individuals such that comparisons of trait selection analysis can be made between different years.

Figure S10 Visualization of quadratic selection differentials for each of the 18 traits

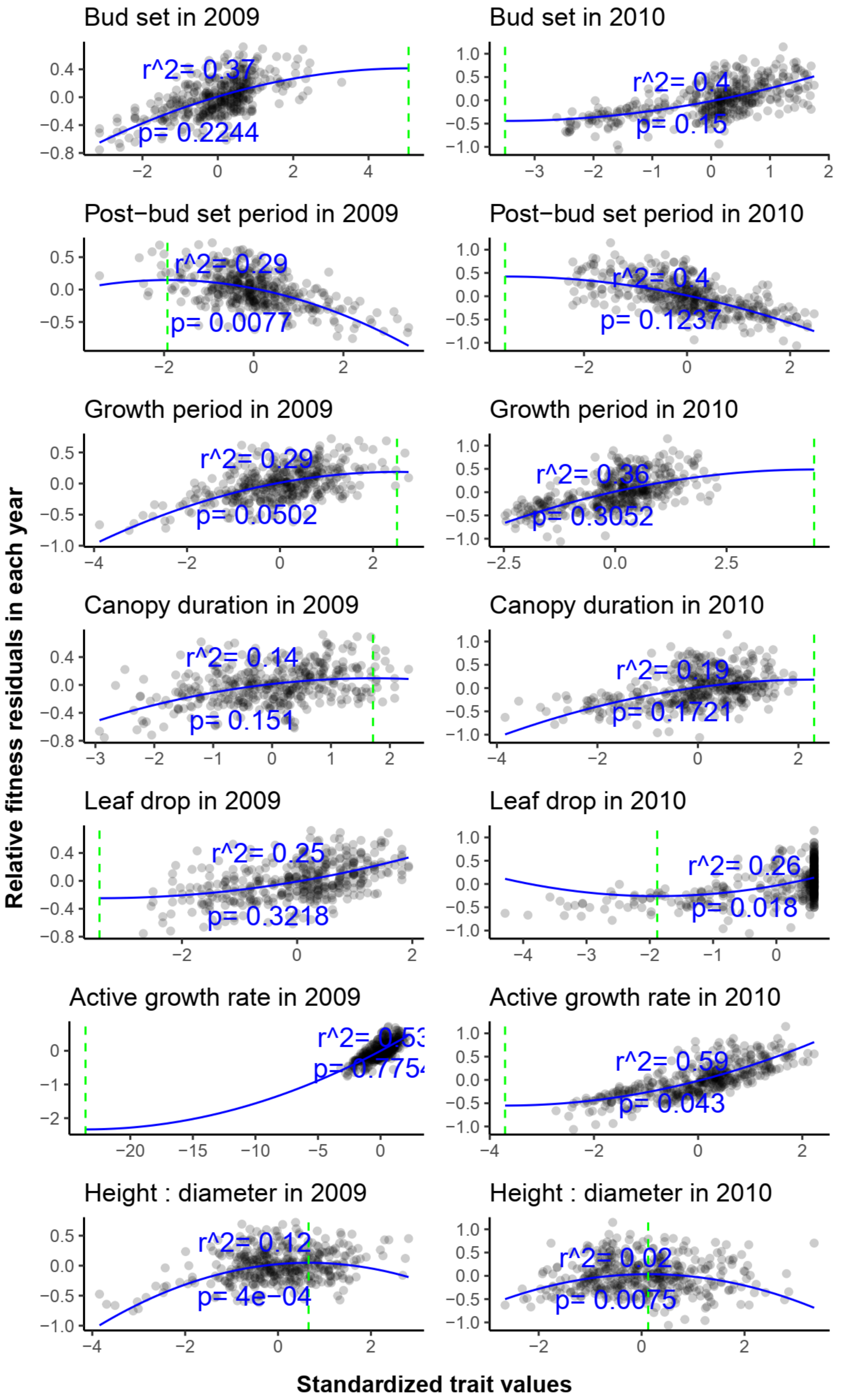

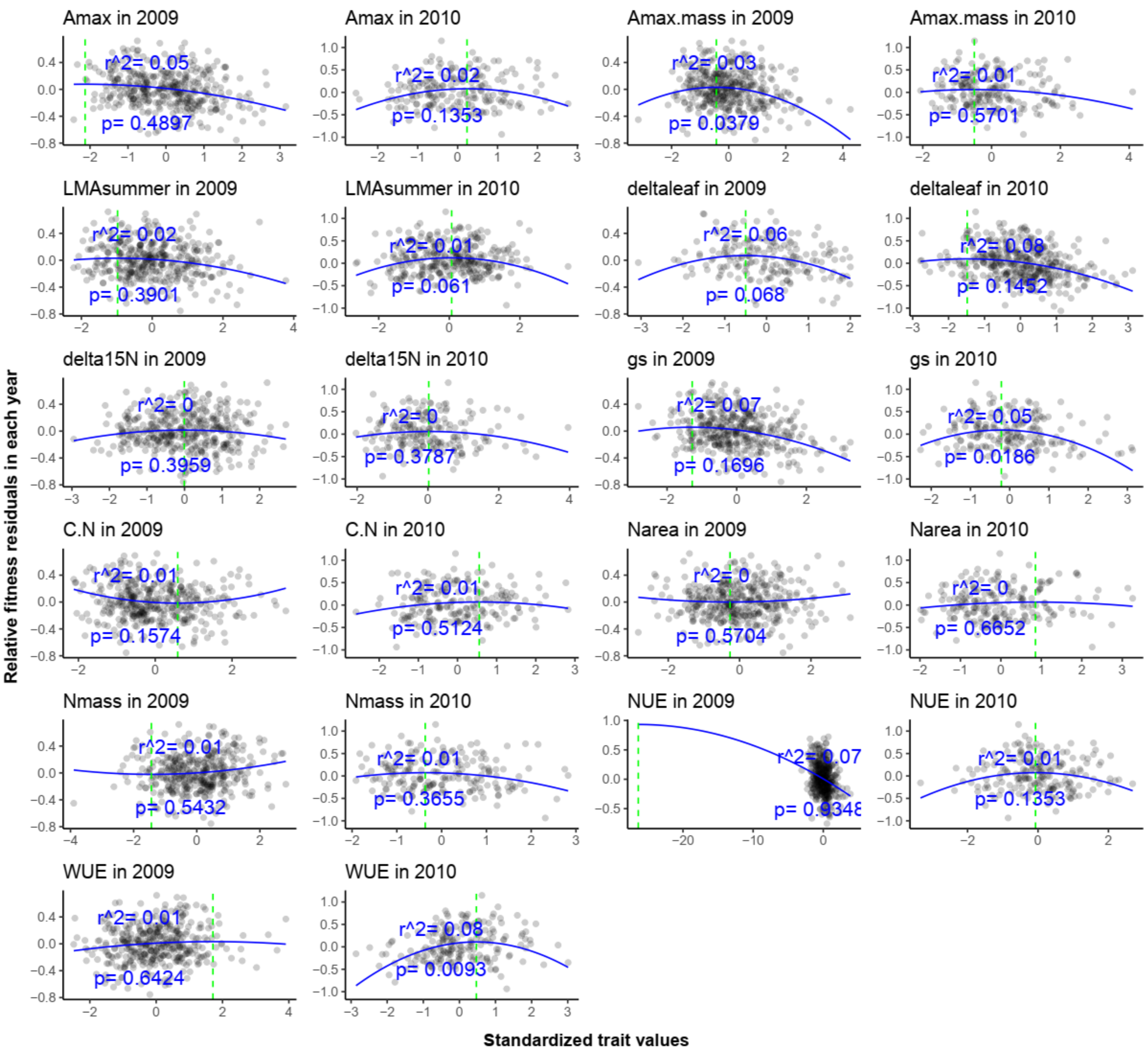

Note:
Dashed green lines depicted peaks in quadratic selection for each trait.
P-values were calculated using *t* tests for gamma (Note S5 for model information; individual relative fitness *residuals* were used instead) and were corrected using the sequential Bonferroni method.
r^2 gave how the quadratic function summarizes the data.
Relative fitness residuals (details in Note S6) were used as fitness for individuals such that comparisons of trait selection analysis can be easily made by different years.

Figure S11 Present-day *P. trichocarpa* suitability scores across its distribution range

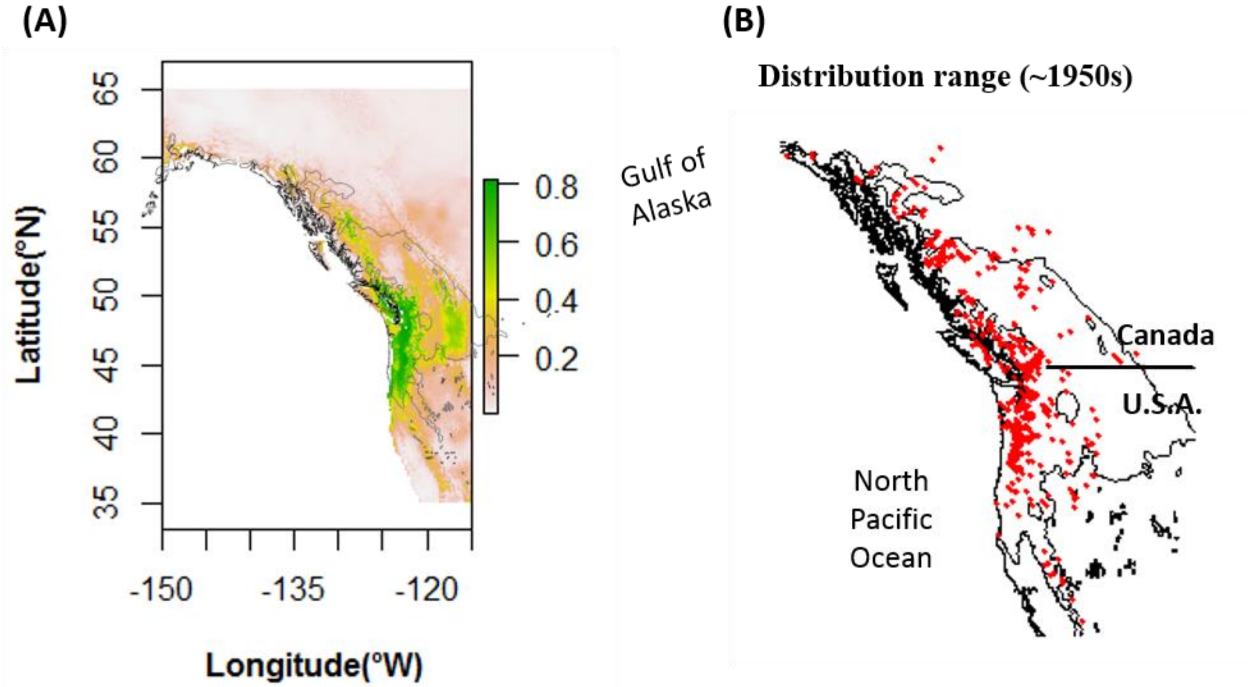

(A) Present-day niche suitability scores across the *P. trichocarpa* distribution range modeled using 524 georeferenced sites on MaxEnt v.3.4.1 at https://biodiversityinformatics.amnh.org/open_source/maxent/. The scores indicate logistic probability of presence ranging from 0 to 1. The suitability scores were first averaged over 20 replicates in each model, and then used to calculate the ratios between models. (B) The species distribution in the 1950s regraphed from (Little 1971) with 524 red dots representing georeferenced occurrence locales used for the environmental niche modeling. This distribution range outline was also manually overlain on the graph (A).

Figure S12 Individual phylogeny based on individual Euclidean genetic distance

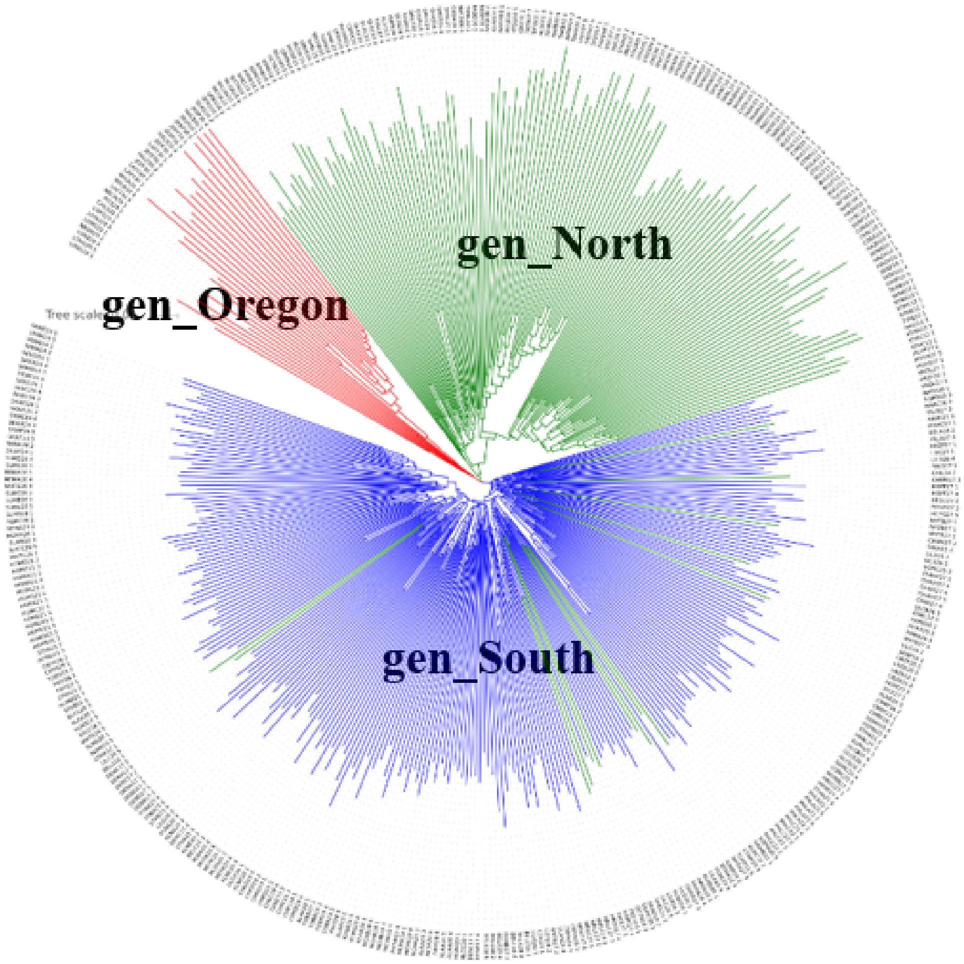

Note: Branches in green, blue, and red indicate individuals from northern, southern, and Oregon demes, respectively. The phylogenetic tree was constructed using Neighbor-Joining (NJ) algorithm (Saitou & Nei 1987) on MEGA v7.1 (Kumar *et al.* 2016) and 1,000 replicates for bootstrap confidence analysis were employed for genetic distance calculation and phylogenetic construction. Scale bar corresponds to the number of substitutions per site.

Supplementary Methods

Note S1 Trait measurements

Seasonal canopy events were recorded directly from observations of trees and calculated additional traits based on phenological date information. The Julian dates of phenological events were recorded for each tree, including bud break, final bud set and leaf drop. Phenology events were marked using visual observations of the terminal bud on the main bole or canopy as a whole (Soolanayakanahally *et al.* 2013). The full canopy duration was calculated as the time from bud break to leaf drop. Active growth period and post-bud set periods were calculated based on days from bud break to final bud set, and from bud set to leaf drop, respectively.

General growth was estimated at the end of each season through measuring tree height (H; cm) and basal diameter at 10 cm from the ground (D; cm). Height : diameter ratios for each year were calculated (H : D; cm cm^{-1}). Bole volume (cm^3) was calculated via assuming a cone shape: $1/3 \times \pi \times (D/2)^2 \times H$. Both height and volume were determined for each individual year. Active growth rate (cm d^{-1}) was determined from yearly height gain divided by the growth period. Note that in 2008, only height was measured and we were unable to calculate biomass increase for 2009. Therefore, we used height gain instead of biomass gain as a proxy for fitness throughout this study.

All ecophysiology traits measurements were taken during the seasons of 2009-2010 before bud set. 11 traits were assessed using gas exchange, and subsequent tissue sampling, on fully exposed, upper canopy leaves taking measurements once from each individual throughout May - August. All sampling was conducted on clear, sunny days between 8:00-14:00 using either a LI-COR 6400 or LI-COR 6400 XT portable infrared gas exchange system (LI-COR Biosciences, Lincoln, NE, USA). Three gas exchange traits were directly measured including maximum photosynthetic rate (A_{max}; µmol CO_2 m^{-2} s^{-1}), stomatal conductance (g_s; mol H_2O m^{-2} s^{-1}), and instantaneous water-use efficiency as determined by photosynthetic rate over transpiration under constant vapor pressure deficit (WUE; µmol CO_2 $mmol^{-1}$

H_2O). After gas exchange sampling, two leaf tissue discs (61 mm^2) were taken using a standard, hand-held punch from an upper canopy leaf on each tree. Samples were oven dried at 50°C for 48h and weighed to determine leaf mass per unit area (LMA; mg cm^{-2}) and to calculate photosynthetic rate per unit dry mass ($A_{max/mass}$; µmol CO_2 g^{-1} s^{-1}). Between 2 and 2.5 mg of dried tissue was analyzed for carbon (C) and nitrogen (N) content and stable isotope ratios ($\delta^{13}C$, $\delta^{15}N$, respectively; ‰) at the UC Davis Stable Isotope Facility (Davis, CA, USA). Based on these data, C to N ratio (C : N; mg mg^{-1}), leaf N content per unit area (N_{area}; mg mm^{-2}) and per unit dry mass (N_{mass}; mg mg^{-1}) and photosynthetic N-use efficiency (NUE; µmol CO_2 g^{-1} N s^{-1}). $\delta^{13}C$ values were used with correction for sampling date (McKown *et al.* 2013) to obtain net discrimination (Δ_{leaf}; ‰) as a proxy measurement for time-integrated WUE.

Note S2 Climate data over the period of trait measurements (2008-2010)

Climate data of 2008-2010 were obtained using ClimateNA ver.6.2.0 (Wang *et al.* 2016). ClimateNA (Wang *et al.* 2016) is a standalone software application that extracts and downscales gridded (4 × 4 km) monthly climate data for a given year or a time period from Parameter-elevation Regressions on Independent Slopes Model (PRISM©) (Daly *et al.* 2008) and WorldClim (Hijmans *et al.* 2005) to scale-free point locales. For consistency, we also extracted mean annual temperature (MAT) and mean annual precipitation (MAP) normals for 1961-1990 for tree origin sites from ClimateNA at http://www.climatewna.com/ instead of from WorldClim at http://www.worldclim.org/bioclim which was used to construct a species distribution model (i.e., bioclimatic variables; Note S8).

Note S3 REML-linear mixed model to partition variance in the 18 traits

To evaluate plasticity of phenology, biomass and ecophysiology traits at the population level and over time, we utilized univariate residual maximum likelihood (REML)-linear mixed models (LMMs) to partition variance in each of 18 study traits. The LMM is written in matrix notation as,

$$\mathbf{Y = X}\beta \mathbf{+ Zu + \varepsilon, u \sim \mathcal{N}(0, G)} \text{ and } \mathbf{\varepsilon \sim \mathcal{N}(0, R)} \qquad \text{Eqn.1}$$

where **X**β is fixed effects, **Zu** represents random effects with a variance-covariance matrix **G**, and **ε** is the residual error with an error covariance matrix **R**. In the model, errors are split into two levels (**Zu** and **ε**) and both levels were assumed to be identically independently distributed (i.e. iid normal). We specified separate models for each trait with fixed effects (**X**β) including temporal environment in a common garden (i.e., Year), Population (nested in Year), and their interactions. Moreover, we included a random factor (**Zu**), 'Genetics', estimated by the average Euclidean genetic distance between genotypes using SNP data. Specifically, we modeled each trait as,

Trait = Year + Population (Year) + Year × Population + Genetics + error

$$\boldsymbol{z_{ijk} = \mu + \alpha_i + \delta_{ij} + \alpha_i \times \delta_{ij} + g_{ijk} + \varepsilon_{ijk}}$$

where $\boldsymbol{\mu}$ is the mean trait value; $\boldsymbol{\alpha_i}$ is the main effect of the ith year; $\boldsymbol{\delta_{ij}}$ is the fixed effect of the jth population nested in the ith year; $\boldsymbol{g_{ijk}}$ is the Genetic relationship for the kth genotype in the jth population in the ith year; and $\boldsymbol{\varepsilon_{ijk}}$ is the random error deviation. Errors were assumed to be iid normal. Genetics calculated as average Euclidean genetic distance among genotypes was the only random-intercept term and the other terms were fixed effects. To verify whether our data conformed to the assumptions for ANOVA, we visually inspected the residuals of each model using Q-Q plot and by plotting them against fitted values. All LMMs were performed using the 'lmer' function of the R packages lme4 and lmerTest (Bates *et al.* 2015; Kuznetsova *et al.* 2017). We reported *F*-values and *P*-values for fixed effects and χ^2-values and *P*-values for random effects after the sequential Bonferroni correction.

To quantify plasticity at the population level and between years, we estimated ordinary least-squares (OLS) mean trait values for the fixed effects (Year, Population, and Year × Population). The OLS estimation is equivalent to the same fixed effects terms in an OLS model expressed as,

$$\mathbf{Y} = \mathbf{X}\beta + \boldsymbol{\varepsilon},\ \boldsymbol{\varepsilon} \sim \mathcal{N}(\mathbf{0}, \mathbf{R}) \qquad \text{Eqn.2}$$

The only difference between the LMM and the OLS model is that the latter has no random effects (**Zu**). Technically, we directly extracted OLS values for a specific fixed effect from the mixed model by using the 'lsmean' function of the R package lsmeans. In addition, we performed Spearman's rank correlation tests of the OLS means for the Population fixed effect to verify which traits are highly correlated. OLS mean trait values for Year, Population, and Year × Population fixed effects were used to quantify plasticity between years.

****

• <u>R code for this analysis</u> (take the "Bud set" trait as an example):

```
MLM.Budset=lmer(Budset~Population*Year+(1|Genetics),REML = TRUE,
                data=filter(dat,!is.na(Budset)))
dat$yhat.MLM.Budset1=fitted(MLM.Budset,level=0)#marginal
dat$yhat.MLM.Budset2=fitted(MLM.Budset,level=1)#conditional
dat$res.MLM.Budset1=fitted(MLM.Budset,level=0)
dat$res.MLM.Budset2=fitted(MLM.Budset,level=1)
#get diagnostic plots for the model conditional means
par(mfrow=c(2,2),mai=c(0.6,0.6,0.6,0.6),cex=0.55)
plot(dat$yhat.GxE.Year.Budset2,dat$res.GxE.Year.Budset2,
     main = "Conditional residual plot",xlab="yhat",ylab="residual")
qqnorm(dat$res.MLM.Budset2,main = "Conditional normality plot")
hist(dat$res.MLM.Budset2,breaks=8,density=10,col="green",border="black",main=
"Conditional residuals distribution")
plot(dat$Budset,dat$res.MLM.Budset2,main = "Subject-specific fitted line
plot",ylab="yhat",xlab="Budset")
#note that it's not useful to plot model marginal means as they are the
mixture of two level errors (i.e. Zu + epsilon).
```

Note S4 Estimating directional selection in each year

To assess both direct and indirect selection on the 18 traits, we calculated selection differentials of each trait in each of the two years, that is, relative fitness as a function of a given trait in a given year. We performed phenotypic selection analysis separately for each trait in each year using linear mixed models. Let $\boldsymbol{z_{ik}}$ be the trait value of the *k*th genotype in the *i*th year, centered and standardized as Z-scores such that mean and variance within each year equal to 0 and 1, respectively, to avoid measurement dimension effects. Within-year relative fitness of each genotype $\boldsymbol{W_{ik}}$ was calculated as the fitness of the *k*th genotype in the *i*th year divided by the mean fitness of a specific year, such that the mean $\boldsymbol{W_{ik}}$ in each year equals to 1. In short, standardized traits and relative fitness were calculated as,

```
An individual’s standardized trait value = (an observed trait value –
mean(trait values of a year))/sd(trait values of that year)
```

```
An individual’s within-year relative fitness = (an individual’s
fitness)/mean(fitness values of a year)
```

We fitted the linear mixed model as,

Individual relative fitness = Trait + Genetics + error

$$\boldsymbol{W_{lk} = \mu + \beta * z_{lk} + g_l + \varepsilon_{lk}}$$

where $\boldsymbol{W_{lk}}$ is the relative fitness of the *k*th genotype in the *l*th genetic group; $\boldsymbol{\mu}$ is the mean relative fitness; $\boldsymbol{z_{lk}}$ is standardized trait value of the *k*th genotype in the *l*th genetic group; $\boldsymbol{\beta}$ is the within-year linear selection differential or linear regression coefficient of relative fitness onto standardized trait values; $\boldsymbol{g_l}$ is the random-intercept term (i.e. ‘Genetics’) for the *l*th genetic group (NOT at the genotype level); and $\boldsymbol{\varepsilon_{lk}}$ is the random error deviation. This model was fitted for 36 times: once for each of the 18 traits in each of the two years, producing 18 linear selection differentials (β’s). It is worth noting that the average Euclidean genetic distance among individuals (i.e. the random effect ‘Genetics’) was rounded up such that individuals belonged to several groups (i.e. *l*’s here) and in each group, individuals had high genetic similarities.

As model residuals often do not satisfy the assumptions of ANOVA, we used a permutation test to determine statistical significance. We compared the observed *F*-value to the distribution of *F*-values generated by permuting standardized trait values among genotypes within year and then re-fitting the linear mixed model 5,000 times. The regression coefficient β is the selection differential for one trait in a year. A significant β indicates nonzero linear selection. The signs and magnitudes of the β's indicate the direction and strength of linear selection on each trait in each year.

Above we fitted the mixed model separately for each year to estimate selection differentials. Furthermore, we tested whether these selection differentials varied between years (i.e. whether selection was temporally variable) by pooling the relative fitness and phenotype data from both years and fitting a mixed-effects ANCOVA model with an additional interaction term:

Individual relative fitness = Year + Trait + Year × Trait + Genetics + error

$$\boldsymbol{W_{ilk} = \mu + \alpha_i + \beta * z_{ilk} + (\alpha * \beta)_i * z_{ilk} + g_{il} + \varepsilon_{ilk}}$$

where $\boldsymbol{W_{ilk}}$ is the relative fitness of the kth genotype in the lth genetic group in the ith year; $\boldsymbol{\mu}$ is the mean relative fitness; $\boldsymbol{\alpha_i}$ is the main effect of the ith year; $\boldsymbol{z_{ilk}}$ is standardized trait value of the kth genotype in the lth genetic group in the ith year; $\boldsymbol{\beta}$ is the regression coefficient of $\boldsymbol{W_{ilk}}$ onto $\boldsymbol{z_{ilk}}$ across years (i.e. average selection differential); $(\boldsymbol{\alpha * \beta})_i$ is the deviation attributed to the regression of $\boldsymbol{W_{ilk}}$ onto $\boldsymbol{z_{ilk}}$ in the ith year (i.e. interaction between year and selection differential); $\boldsymbol{g_{il}}$ is the random-intercept term (i.e. 'Genetics') for the lth genetic group in the ith year; and $\boldsymbol{\varepsilon_{ik}}$ is the random error deviation. A nonzero $(\boldsymbol{\alpha * \beta})_i$ term (i.e. a significant year-by-trait interaction) indicates *heterogeneous directional selection* between years, where $\boldsymbol{\beta}$ alone indicates *average directional selection*. We fitted this model 18 times, once for each of the 18 traits.

Selection differentials allow us to estimate the combined impact of direct and indirect selection on each trait. However, for thoroughness we also calculated selection gradients in each year, that is, selection on each trait after controlling for selection on the other traits within one multiple linear

regression. This means the selection gradient represents a hypothetical scenario in which direct selection acts on one target trait while assuming indirect selection on the others.

Individual relative fitness = ∑(traits) + Genetics + error

$$\boldsymbol{W_{lk}} = \boldsymbol{\mu} + \sum_{\boldsymbol{n=1}}^{\boldsymbol{N=18}} \boldsymbol{\beta_n} * \boldsymbol{z_{nlk}} + \boldsymbol{g_l} + \boldsymbol{\varepsilon_{lk}}$$

where $\boldsymbol{W_{lk}}$ is the relative fitness of the kth genotype in the lth genetic group; $\boldsymbol{\mu}$ is the mean relative fitness; $\boldsymbol{z_{nlk}}$ is the standardized trait values of the kth genotype in the lth genetic group for a total of 18 traits (N = 18); $\boldsymbol{\beta_n}$ is the selection gradients or partial regression coefficients of relative fitness onto corresponding trait values $\boldsymbol{z_n}$; $\boldsymbol{g_l}$ is the random effect of the lth genetic group; and $\boldsymbol{\varepsilon_{lk}}$ is the residual error. We fitted this model twice, once for each of the two years.

Note S5 Estimating quadratic selection in each year

As stabilizing or disruptive selection could also affect the fitness consequences of plasticity (i.e. movement of trait values either toward or away from optimum values in each year), we performed quadratic selection analysis for each trait in each year using a modified linear mixed model. Briefly, compared with Eqn.2 in Note S3, a quadratic fixed effects term was added to the LMM, written as, $\mathbf{Y} = \mathbf{X}\beta + \mathbf{X}^2\gamma + \mathbf{Zu} + \boldsymbol{\varepsilon}$, where $\mathbf{X}\beta$ and $\mathbf{X}^2\gamma$ are fixed effects, **Zu** represents random effects (design matrix **Z** and coefficient **u**), and $\boldsymbol{\varepsilon}$ is the residual error. Specifically, we fitted the linear mixed model as,

Individual relative fitness = Trait + (Trait)^2 + Genetics + error

$$\boldsymbol{W_{lk}} = \boldsymbol{\mu} + \boldsymbol{\beta} * \boldsymbol{z_{lk}} + \boldsymbol{\gamma} * \boldsymbol{z_{lk}^2} + \boldsymbol{g_l} + \boldsymbol{\varepsilon_{lk}}$$

where $\boldsymbol{W_{lk}}$ is the relative fitness of the kth genotype in the lth genetic group; $\boldsymbol{\mu}$ is the mean relative fitness; $\boldsymbol{z_{lk}}$ is standardized trait value of the kth genotype in the lth genetic group; $\boldsymbol{\beta}$ is the within-year linear selection differential or linear regression coefficient of relative fitness onto standardized trait values; $\boldsymbol{\gamma}$ is the quadratic regression coefficient of relative fitness onto standardized trait values; $\boldsymbol{g_l}$ is the random-intercept term (i.e. 'Genetics') for the lth genetic group; and $\boldsymbol{\varepsilon_{lk}}$ is the random error deviation. This model was fitted for 36 times: once for each of the 18 traits in each of the two years, producing 36 linear selection differentials (γ's). We doubled each γ value to accurately reflect the strength of quadratic selection (Stinchcombe *et al.* 2008). A significant γ regression coefficient indicates nonzero quadratic selection; negative values are taken as evidence of stabilizing selection, while positive values indicate disruptive selection.

Note S6 Estimating relative fitness residuals in each year

To better compare the degree of selection between years, the within-year relative fitness residuals of all individuals were regressed onto standardized trait values. We estimated residuals of relative fitness using the random-effect model:

Individual relative fitness = Genetics + error

$$\boldsymbol{W_{lk}} = \boldsymbol{\mu} + \boldsymbol{g_l} + \boldsymbol{\varepsilon_{lk}}$$

where $\boldsymbol{W_{lk}}$ is the relative fitness of the kth genotype in the lth genetic group; $\boldsymbol{\mu}$ is the mean relative fitness; $\boldsymbol{g_l}$ is the random-intercept term (i.e. 'Genetics') for the lth genetic group; and $\boldsymbol{\varepsilon_{lk}}$ is the random error deviation. The residuals were extracted from the model by using `resid` function in R.

Note S7 Analysis of the joint evolution of traits and source niche climate

BayesTraits (Pagel & Meade 2007) analyzes continuous phenotypes using a phylogenetically generalized least-squares approach under the assumption of Brownian motion, estimating correlation coefficients and measures of support for correlated evolution between variables. A phylogenetic tree for the individuals was constructed using individual Euclidean genetic distance (Fig. S12). For each set of phylogenetic tree and phenotypes or climate variables, we assessed a model using the continuous function under an MCMC setting, estimating the log marginal likelihood using the stepping stone method [SS; (Xie *et al.* 2011)] with 100 stones and 1,000 iterations per stone. We estimated the log Bayes factor (log*BF*) for the dependent model (correlation between variables allowed) against the independent model (all correlations fixed to zero) as twice the difference between the estimated log marginal likelihoods, that is, $\log BF = 2 \times (SS_{dep} - SS_{indep})$, where SS_{dep} and SS_{indep} are the estimated log marginal likelihoods for the dependent and independent models, respectively. We interpreted comparisons where $\log BF > 2$, 5 or 10 as having weak, moderate, or strong support, respectively. This analysis was performed on all 120 trait-climate combinations for all the populations. Based on $\log BF > 5$ as moderate support in favor of the model with a higher marginal likelihood (Kass & Raftery 1995), we accounted for multiple comparisons by identifying those comparisons with $\log BF > 5 + \log(N)$, where N is the number of model comparisons performed. For the 120 phenotype-climate combinations, the threshold for a significant model corresponds to $\log BF > 9.8$. In this analysis, all 20 phenotypes were averaged over the two years and then natural-log-transformed, and all six climate variables were Z-transformed. In addition, we run the analysis for latitude separately such that we can echo back to our findings showing that the variation of study traits covaries with latitude (e.g., Fig. 2 in the main text).

Note S8 Niche suitability identifying key climate variables for species persistence

Previously, we used 524 *P. trichocarpa* georeferenced records from the literature and herbaria to predict present-day niche suitability across this species distribution range using MaxEnt v.3.4.1 (Fig. S11). This software employs a machine-learning approach to identify the probability that a species has occurred based on known occurrence sites (Phillips *et al.* 2006). Our species distribution modeling had a relatively high accuracy (area under the curve [AUC] = 0.89; standard deviation [SD] = 0.03) (unpubl. work). Moreover, the climate variables that most contributed to the model prediction was mean annual precipitation (40.2%) followed by mean annual temperature (38.7%). Note that after a reduction of collinearity ($|r| > 0.7$) for climatic variables based on 524 niches across this species distribution range, a total of six bioclimatic variables from WorldClim at http://www.worldclim.org/bioclim were used for modeling. These variables include Mean Annual Temperature (MAT; BIO1), Mean Diurnal Range (DiurnalRange; BIO2), Mean Temperature of Wettest Quarter (TWettestQtr; BIO8), Mean Annual Precipitation (MAP; BIO12), Precipitation Seasonality (PSeasonality; BIO15) and Precipitation of Warmest Quarter (PWarmestQtr; BIO18).

Supplementary References

Bates D, Machler M, Bolker BM, Walker SC (2015) Fitting linear mixed effects models using lme4. *Journal of Statistical Software* **67**, 1-48.

Daly C, Halbleib M, Smith JI*, et al.* (2008) Physiographically sensitive mapping of climatological temperature and precipitation across the conterminous United States. *International Journal of Climatology* **28**, 2031-2064.

Geraldes A, Farzaneh N, Grassa CJ*, et al.* (2014) Landscape genomics of *Populus trichocarpa*: The role of hybridization, limited gene flow, and natural selection in shaping patterns of population structure. *Evolution* **68**, 3260-3280.

Hijmans RJ, Cameron SE, Parra JL, Jones PG, Jarvis A (2005) Very high resolution interpolated climate surfaces for global land areas. *International Journal of Climatology* **25**, 1965-1978.

Kass RE, Raftery AE (1995) Bayes factors. *Journal of the American Statistical Association* **90**, 773-795.

Kumar S, Stecher G, Tamura K (2016) MEGA7: Molecular evolutionary genetics analysis version 7.0 for bigger datasets. *Molecular Biology and Evolution* **33**, 1870-1874.

Kuznetsova A, Brockhoff PB, Christensen RHB (2017) lmerTest Package: Tests in linear mixed effects models. *Journal of Statistical Software* **82**, 1-26.

Little EL (1971) *Atlas of United States trees* US Department of Agriculture, Forest Service, Washington, DC.

McKown AD, Guy RD, Azam MS, Drewes EC, Quamme LK (2013) Seasonality and phenology alter functional leaf traits. *Oecologia* **172**, 653-665.

Pagel M, Meade A (2007) BayesTraits. Computer program and documentation available at http://www.evolution.rdg.ac.uk/BayesTraits.html.

Phillips SJ, Anderson RP, Schapire RE (2006) Maximum entropy modeling of species geographic distributions. *Ecological Modelling* **190**, 231-259.

Saitou N, Nei M (1987) The neighbor-joining method: A new method for reconstructing phylogenetic trees. *Molecular Biology and Evolution* **4**, 406-425.

Soolanayakanahally RY, Guy RD, Silim SN, Song MH (2013) Timing of photoperiodic competency causes phenological mismatch in balsam poplar (*Populus balsamifera* L.). *Plant Cell and Environment* **36**, 116-127.

Stinchcombe JR, Agrawal AF, Hohenlohe PA, Arnold SJ, Blows MW (2008) Estimating nonlinear selection gradients using quadratic regression coefficients: Double or nothing? *Evolution* **62**, 2435-2440.

Wang TL, Hamann A, Spittlehouse D, Carroll C (2016) Locally downscaled and spatially customizable climate data for historical and future periods for North America. *PLoS ONE* **11**, e0156720.

Xie W, Lewis PO, Fan Y, Kuo L, Chen MH (2011) Improving marginal likelihood estimation for Bayesian phylogenetic model selection. *Systematic Biology* **60**, 150-160.
